# Cell type-selective disease-association of genes under high regulatory load

**DOI:** 10.1093/nar/gkv863

**Published:** 2015-10-10

**Authors:** Mafalda Galhardo, Philipp Berninger, Thanh-Phuong Nguyen, Thomas Sauter, Lasse Sinkkonen

**Affiliations:** 1 Life Sciences Research Unit, University of Luxembourg, L-1511 Luxembourg, Luxembourg; 2 Biozentrum, University of Basel and Swiss Institute of Bioinformatics, 4056 Basel, Switzerland

## Abstract

We previously showed that disease-linked metabolic genes are often under combinatorial regulation. Using the genome-wide ChIP-Seq binding profiles for 93 transcription factors in nine different cell lines, we show that genes under high regulatory load are significantly enriched for disease-association across cell types. We find that transcription factor load correlates with the enhancer load of the genes and thereby allows the identification of genes under high regulatory load by epigenomic mapping of active enhancers. Identification of the high enhancer load genes across 139 samples from 96 different cell and tissue types reveals a consistent enrichment for disease-associated genes in a cell type-selective manner. The underlying genes are not limited to super-enhancer genes and show several types of disease-association evidence beyond genetic variation (such as biomarkers). Interestingly, the high regulatory load genes are involved in more KEGG pathways than expected by chance, exhibit increased betweenness centrality in the interaction network of liver disease genes, and carry longer 3′ UTRs with more microRNA (miRNA) binding sites than genes on average, suggesting a role as hubs integrating signals within regulatory networks. In summary, epigenetic mapping of active enhancers presents a promising and unbiased approach for identification of novel disease genes in a cell type-selective manner.

## INTRODUCTION

Identification of disease-relevant genes and gene products as biomarkers and drug targets is one of the key tasks of biomedical research. Great progress has been made in diagnosing and treating various diseases over the past decades. Still, a great majority of research is focused on a small minority of genes while over a third of genes remain unstudied (1). Unbiased prioritization within these ignored genes would be important to harvest the full potential of genomics in understanding diseases.

Many databases to catalog disease-associated genes and the nature of their association, such as the Comparative Toxicogenomics Database (CTD) or the Online Mendelian Inheritance in Man (OMIM), have been created (2,3). One of the more comprehensive databases, DisGeNET (4,5), draws from multiple sources as well as text-mining approaches to generate gene-disease networks where genes are associated to diseases by various evidence ranging from altered expression and genetic variation to existing therapeutic association. DisGeNET already links many of the human genes to at least one disease, highlights the multi-genetic background of most diseases and how many genes can be associated to multiple diseases (4,5).

Interestingly, as much as 90% of the human disease-associated genetic variants are located outside of the coding sequences of protein coding genes, suggesting that they affect the regulation of these genes instead (6,7). The active regulatory regions of the genome can be identified in a cell type-specific manner through chromatin immunoprecipitation coupled with deep sequencing (ChIP-Seq) analysis of selected covalent histone modifications such as histone H3 lysine 27 acetylation (H3K27ac; marking active enhancers) and histone H3 lysine 4 trimethylation (H3K4me3; marking open transcription start sites), among others. Indeed, by taking advantage of such epigenomic data produced by the Roadmap Epigenomics Mapping Consortium, Farh *et al*. (8) recently showed that up to 60% of human autoimmune variants are located within active enhancers of immune cells. In particular, the genetic variants seem to coincide with so called super-enhancers or stretch-enhancers, large enhancer regions often associated with key genes and master regulators of cellular identity (9–11). These enhancers function as hotspots with binding sites for multiple transcription factors (TFs) (12) and, within the enhancers, single nucleotide polymorphisms (SNPs) often disrupt these binding sites as shown, for example, for type 2 diabetes variants within islet enhancers (13). However, it remains unclear whether the genes controlled by multiple enhancers and TFs are associated to disease also beyond the genetic variation in their regulatory regions, as could be assumed from their role as regulators of cellular identity.

We have previously shown that metabolic genes regulated by multiple TFs in human umbilical vein endothelial cells (HUVEC) are enriched for genes associated to endothelial relevant diseases in DisGeNET (14). Here we set out to test whether this increased disease-association of genes under high regulatory load (HRL) is a general observation that holds across cell types and genes, and independent of the type of disease-association evidence. Analysis of ChIP-Seq data for 93 TFs across 9 ENCODE cell lines confirms an enrichment for disease-association among the highest regulated genes in all cell types. We find that the TF load of the genes correlates with their enhancer load in the respective cell types and thereby allows the identification of genes under high regulatory load by epigenomic mapping of active enhancers using H3K27ac. Consistently, genes associated with most enhancers are also most enriched for disease-association in all 9 cell lines. To elucidate the power of this approach and to analyze the cell type selectivity of the disease-associations, we perform disease-association enrichment analysis for high enhancer load genes from 139 ChIP-Seq samples of H3K27ac corresponding to 96 different cell types and tissues, with many diseases showing high level of cell type selectivity. Finally, we show that genes under high enhancer load are involved in more Kyoto Encyclopaedia of Genes and Genomes (KEGG) pathways and exhibit higher betweenness centrality in a liver disease gene network than other genes on average, suggesting a central role in integrating multiple signals in biological networks. Consistently, the genes under high regulatory load at the transcriptional level have longer 3′ untranslated regions (3′ UTRs) and contain more microRNA (miRNA) binding sites than other genes, suggesting that they could be under higher regulatory load also at the post-transcriptional level.

Taken together, these results paint a picture of high regulatory load genes as central nodes in biological networks, that are more likely to be associated with human disease, and identifies epigenomic analysis of active enhancers as a tool for cell type-selective prioritization of previously unstudied genes.

## MATERIALS AND METHODS

### Disease-associated genes

Gene-disease association data were retrieved from the DisGeNET Database (GRIB/IMIM/UPF Integrative Biomedical Informatics Group, Barcelona http://www.disgenet.org/ version 2.1, 5th of May 2014). DisGeNET provides gene-disease associations from several public data sources and literature text-mining, with a score ranking associations based on the supporting evidence. A minimum association score of 0.08 was used to select gene-disease associations supported by multiple data sources and to exclude associations that are based solely on text-mining results, resulting in 7428 disease-associated genes, of which 6167 were contained in our background set of 19 238 protein coding genes (Supplementary File 1). Alternatively, a minimum association score of 0.2 characterizes curated disease-associated genes (7110 genes, of which 5853 were in the background set) (gene-disease associations from UNIPROT, ClinVar and CTD human data set, see http://www.disgenet.org/web/DisGeNET/menu/dbinfo). Additionally, as a separate set of high confidence disease genes we used the OMIM database (downloaded from ftp://ftp.omim.org/OMIM/, as of June 2015) (4557 genes of which 3483 were in the background set). For gene set enrichment testing we selected only diseases with at least 15 associated genes, to avoid significant results only due to a very small set size, resulting in 340 diseases (Supplementary File 1) (15). Details about the gene-disease-association types defined in the DisGeNET for each disease are also found in the Supplementary File 1, and they include ‘altered expression’, ‘biomarker’, ‘genetic variation’, ‘post-translational modification’ and ‘therapeutic’. To test whether ‘genetic variation’ was predominantly accounting for disease-association enrichment, we defined the group ‘not genetic variation’ by pooling all disease-associated genes with association evidence other than ‘genetic variation’.

### Background set of protein coding genes and their ‘regulatory domain’

We focused on protein coding genes in the analysis. The NCBI Entrez Gene annotations for ‘protein coding’ genes (Homo_sapiens.gene_info file, ftp://ftp.ncbi.nih.gov/gene/DATA/GENE_INFO/Mammalia/, downloaded on the 13th of May 2014) were used to derive a set of genes serving as ‘background’ for gene set enrichment testing. Their TSS was extracted by intersecting with the RefSeq genes file taken from the UCSC Table Browser (16) (http://genome.ucsc.edu/cgi-bin/hgTables, RefSeq genes, assembly: February 2009 (GRCh37/hg19), on the 13th of May 2014), resulting in 19 238 protein coding genes. In order to associate ChIP-seq peaks to the 19 238 genes, we used the Genomic Regions Enrichment of Annotations Tool (GREAT) (17) to derive a ‘regulatory domain’ for each gene, using the script ‘createRegulatoryDomains’ and the rule ‘BasalPlusExtension’ with default settings (source code from http://bejerano.stanford.edu/help/display/GREAT/Download, May 2014). Chromosome sizes of the human genome assembly hg19 were obtained using the script ‘fetchChromSizes’ from the UCSC BigWig and BigBed tools (18). Supplementary File 1 contains details on the 19 238 protein coding genes used for analysis, including their regulatory domains derived by the GREAT tool as start and end coordinates.

### Data sources and processing

Public ChIP-seq data produced by the ENCODE project (19), the BLUEPRINT Epigenome project (20) and the NIH Epigenomic Roadmap project (21) were downloaded from the ENCODE Data Coordination Center (http://genomebrowser.wustl.edu/encode/) on May 2014, the BLUEPRINT consortium website (http://www.blueprint-epigenome.eu) on July 2014, and NIH Epigenomic Roadmap supplementary website (http://compbio.mit.edu/roadmap) on January 2015, respectively. These data span 93 TFs, the H3K4me3 and the H3K27ac modification marks across 139 samples that comprise 96 tissues or cell types (Supplementary File 2). The ENCODE data were no further processed, while the BLUEPRINT and NIH Epigenomic Roadmap data were filtered to keep only peaks with a minimum fold change and (−log_10_ q-value) of 3.

The H3K4me3 data were used to filter out genes embedded in closed chromatin. A file containing genes with at least one H3K4me3 peak within their transcription start sites (TSS) ±1000 bp was obtained per sample, using the IntersectBed tool from the BEDTools suite (22) to intersect each sample's H3K4me3 data with a file containing RefSeq genes and their TSS ±1000 bp as start and end coordinates. In case of multiple H3K4me3 data files per sample, we considered evidence from one single file sufficient to call the mark present. The H3K27ac data served to map active enhancers. The ENCODE project was the only source of TF data. To select only TFs known to directly bind DNA, we used a list of manually curated TFs (23), 111 of which were included in the ENCODE TFs (Supplementary File 2) and 93 had been assayed in the used cell lines, resulting in the presented numbers of unique TFs assayed per cell line. In case of multiple files of the same TF in a cell line (e.g. different ENCODE data producing labs), a filtering step for keeping only peaks overlapping by at least 1 bp in two thirds of the ‘replicates’ was applied. The intersectBed tool was used to intersect TF or H3K27ac data with the file containing regulatory domains for each gene (see above), requiring a peak to completely fall within the genes regulatory domain in order to assign it to that gene. For each TF, we obtained a list of associated genes and derived the TF load per gene from the total number of associated TFs, across nine ENCODE cell lines (A549, GM12878, H1hESC, HCT116, HeLaS3, HepG2, HUVEC, K562 and MCF7). To obtain the enhancer load per gene, we used the count option of the IntersectBed tool to count the number of H3K27ac peaks falling within the genes regulatory domain. For both TFs and enhancers, we ranked genes based on the regulatory load and subsequently considered only genes with the H3K4me3 mark within ±1000 bp of the TSS. Following the above settings, on average 96% of peaks could be associated to a target gene.

### Gene binning and hypergeometric enrichment tests

In order to group genes based on their regulatory load, we started binning ranked genes by deciles (bins containing 10% of the genes), with a separate group for genes with no associated TFs or enhancers (11 starting bins). Bins were then extended by inclusion of all genes with the same regulatory load as the last gene falling in a bin, excluding cases of genes with equal regulatory load falling in different bins (fewer bins depending on the sample). Top bin genes for each sample can be found in Supplementary File 3. We then performed hypergeometric distribution tests for the enrichment of disease genes among the different regulatory load bins per sample and the 340 DisGeNET diseases with at least 15 genes. For each sample, the ‘population size’ corresponded to the number of genes with the H3K4me3 mark (varying per sample), the ‘number of successes’ being the number of disease genes with the H3K4me3 mark (varying per sample) and the ‘number of draws’ the number of genes having the regulatory load of the bin in case (number of TFs or enhancers). Hypergeometric *P*-values were obtained using the Matlab® hypergeometric cumulative distribution function (hygecdf) and were adjusted for multiple testing with the Benjamini and Hochberg methodology as implemented in the Bioconductor's qvalue package (http://www.bioconductor.org/packages/release/bioc/html/qvalue.html).

### Bicluster of hypergeometric enrichment statistical significance

In order to simultaneously cluster samples and diseases into homogeneous blocks based on the hypergeometric enrichment significance (adjusted −log_10_ *P*-values), the R package ‘blockcluster’ (24) was applied to the matrix (of adjusted −log_10_ *P*-values) from the 139 samples and 174 diseases, after binarization (‘zero’ for −log_10_ *P*-value < 1.301, ‘one’ otherwise) and exclusion of diseases or samples only containing ‘zero’. Shortly, block clustering methods estimate a mixture model from permutations of objects and variables in order to draw a correspondence structure (thereby with certain order variability with repetition). ‘Blockcluster’ requires a predefined number of clusters for the rows and columns, which we fixed at 9 and 7, respectively (here, diseases and samples), in order to minimize redundant clusters. Supplementary File 4 contains the ordering for diseases and samples and their clusters (color shades), as obtained with the ‘blockcluster’ package.

### Identification of super-enhancer genes

NIH Roadmap epigenomics raw data were downloaded from the GEO ftp site (ftp://ftp.ncbi.nlm.nih.gov/pub/geo/DATA/roadmapepigenomics/by_experiment/) on May 2015, selecting data for all three from H3K4me3, H3K27ac and Input, resulting in 35 samples. These included bed files of reads aligned onto the hg19 human genome assembly using Pash 3.0 read mapper (http://egg2.wustl.edu/roadmap/web_portal/processed_data.html). As the raw data contained sample names and the processed data used for the high regulatory load genes analysis contained sample IDs, mappings between the two were manually obtained based on descriptions from the original data sources (http://egg2.wustl.edu/roadmap/web_portal/meta.html). Next, the software HOMER (version 4.7, 25th of August 2014) (25) was used for super-enhancer calling on the H3K27ac bed files from each sample, pooling samples from the same origin, with default setting except the local fold change option (-L) which was set to 0 as recommended by the authors for super-enhancer analysis, resulting in the obtainment of the chromosome, start and end coordinates of super-enhancer peaks. We then used the IntersectBed tool from the BEDTools suite (22) and the genes ‘regulatory domain’ file obtained with GREAT (see previous descriptions) to derive a set of super-enhancer-associated genes per sample. These genes were subsequently used for testing the enrichment for disease-association using the hypergeometric distribution, as previously described.

### Analysis of the RNA-seq data

Data were downloaded from http://egg2.wustl.edu/roadmap/web_portal/processed_data.html#RNAseq_uni_proc on June 2015, taking the file ‘57epigenomes.RPKM.pc’ containing the RPKM (reads per kilobase per million mapped reads) for 57 samples, 38 of which were also in the set of 139 samples used for the analysis of high regulatory load genes. Conversion of ENSEMBL IDs to ENTREZ GENE IDs was done using the Bioconductor package ‘biomaRt’ (26), resulting in expression data for 18 220 ENTREZ GENE IDs across samples, out of which 18 181 were included in our background set of 19 238 protein coding genes. For each of the 38 samples, genes were ranked based on expression. Since the set of genes for high regulatory load and expression is not the same, we defined the top bin of highly expressed genes to contain the same number of high regulatory load genes in each sample, triplicating this number for the 30% top bins of expression. The 50% and 90% top expression bins were obtained relative to the total number of genes for which there was expression data (18 181).

### KEGG pathway enrichment testing

KEGG (27) pathways were used to test whether high regulatory load genes appear in more pathways than expected. The list of KEGG pathways was obtained through the REST-style KEGG API from http://rest.kegg.jp/list/pathway/hsa, resulting in 282 pathways with at least one gene. KEGG pathways were downloaded and gene info per pathway was obtained using the R/Bioconductor package ‘KEGGprofile’. The average number of pathways per KEGG gene (total of 6822 genes in all KEGG pathways), per high regulatory load gene (differing from sample to sample) or based on a random selection of an equal number of genes as the high regulatory load genes for each sample (10 000-fold) was calculated. Supplementary File 4 contains the results obtained for each sample. A *P*-value (≤0.05 was considered significant) was calculated from this re-sampling test based on the probability to get at least the same average number of pathways per KEGG gene in random selections as obtained for the high regulatory load genes.

### Constructing a liver disease gene network

The list of liver diseases was curated from the Medical Subject Headings (MeSH) database (http://www.ncbi.nlm.nih.gov/mesh/). The MeSH database is the National Library of Medicine's controlled vocabulary thesauruses consisting of sets of terms structured in a hierarchical form that facilitates searching at different levels. We curated 137 liver diseases. Based on the obtained list of liver diseases, 847 genes related to liver diseases (liver disease genes in short) were extracted from the Comparative Toxicogenomics Database (CTD) database (28). We considered only curated disease-gene associations to increase the reliability of the liver disease gene data. The construction of liver disease gene network was carried out by extracting human protein interactions published in the Human Protein Reference Database (HPRD) (29). The HPRD database contains manually curated protein interactions from literature and is one of the most well-known human protein interaction databases.

The final liver disease gene network of interest consisted of the liver disease genes and their neighbors (nodes), and their direct interactions (edges). In this study, we took into account one-step neighbors. The network was undirected and unweighted because we considered binary interactions. We obtained a network of 3775 genes and 8278 interactions. To unravel the role of genes in the network, we calculated betweenness centrality for each gene and compared the average betweenness centrality of the high regulatory load genes to that of all genes or all genes except those under high regulatory load. Betweenness shows the bridge role of a gene for other genes in the network (30). For each node *v* in the network, we computed the total number of shortest paths from node *s* to node *t*, called *d(s,t)* and the number of those paths that pass through *v*, called *d(s,v t)*, and then ratio *d(s,v, t)/d(s,t)* was calculated. These steps were repeated for all pairs of node *s* and node *t* in the network. The overall betweenness centrality of a node *v* is obtained by summing up those ratios. Betweeness *B(v)* of a node *v* is defined as following:

(1) \begin{equation*} B(v) = \sum\limits_{s \neq v \neq t}{d(s,v,t ) \over d (s,t)} \end{equation*}

### 3′ UTR length and miRNA binding site analysis

Annotation data on 5′ UTR, CDS, 3′ UTR, spliced as well as unspliced transcript length for human mRNA genes was obtained from Biomart (Ensembl Genes 78). Transcripts lacking proper UTR annotation were filtered out. In cases where multiple transcripts correspond to one gene ID, a representative member was randomly chosen. A background set, consisting of 16 307 genes, was used for all comparisons. In order to test the hypothesis, that highly regulated genes tend to have longer 3′ UTRs, we compared in all 139 samples the length of 3′ UTRs, CDS, spliced as well as unspliced transcript length of the high enhancer load genes with the background set with the Kolmogorov-Smirnov test, testing if the background set is smaller than the test set. In order to correct for multiple testing, Bonferroni correction was used, with a significance level ≤0.0003597122 (0.05/139).

Predicted target sites for conserved miRNAs were obtained from TargetScan 6.2 (31). The target site count per 3′ UTR were summed up, resulting in an average site count per transcript. In cases where a site in the 3′ UTR was assigned to multiple miRNAs, it was counted only once.

## RESULTS

### Genes under high regulatory load from multiple transcription factors are enriched for disease-association across cell types

Our previous work on regulation of metabolic genes in human adipocytes and human primary macrophages has uncovered that combinatorial control by multiple regulators is in particular occurring at genes associated to key nodes such as entry points of the metabolic networks and at genes that are often disease-related (14; Pires Pacheco *et al*., under revision). Moreover, analysis of metabolic genes controlled by multiple TFs in HUVEC cells revealed consistent enrichment for endothelial disease-relevant genes among the genes under the highest regulatory load (14).

**Figure 1.**
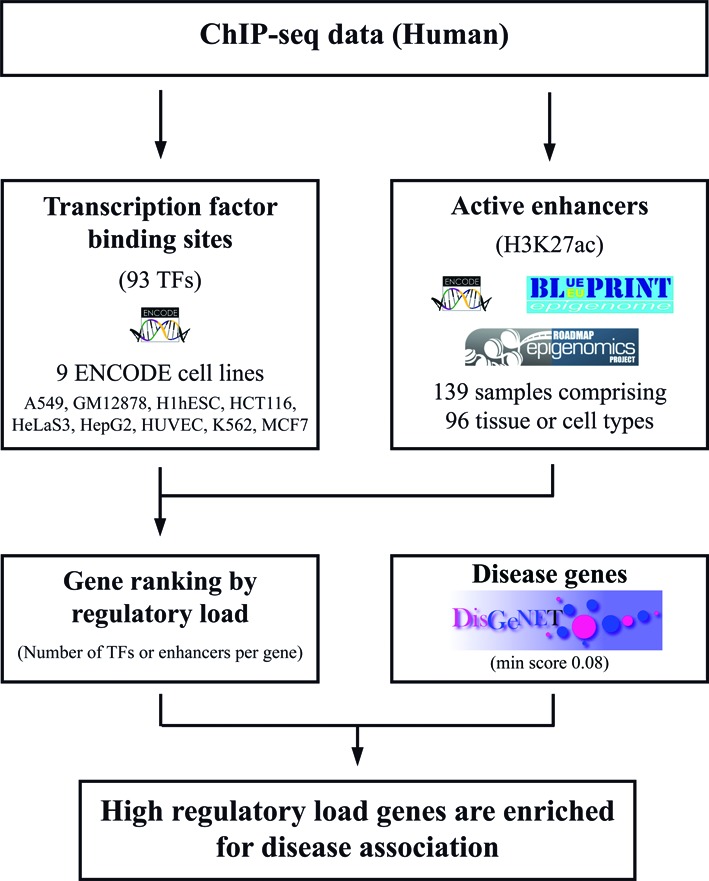
The workflow of the disease-gene enrichment analysis. Processed ChIP-seq data (bed files) from 93 transcription factors (TFs), H3K27ac and H3K4me3 across 139 sample sets were downloaded from the ENCODE (19), NIH Epigenomic Roadmap (21), and BLUEPRINT Epigenome (20) projects (see Supplementary File 2 for additional details). The H3K27ac was used as a mark for active enhancers. The GREAT tool (17) was used to derive a ‘regulatory domain’ for each protein coding gene (‘BasalPlusExtension’ rule with default settings) and the regulatory load per gene was obtained from the number of TF or enhancer peaks falling within the genes regulatory domain. Genes within closed chromatin regions (without the H3K4me3 mark within ±1000 bp from the TSS) were ignored. Gene-disease associations for 340 diseases with at least 15 genes were based on the DisGeNET database (requiring a minimum association score of 0.08), for a total of 7428 disease genes (4,5). The 19 238 protein coding genes (including 6167 of the disease genes) in our background set were grouped into comparable sized bins based on the regulatory load per sample. These ‘regulatory load’ bins were used for testing disease association enrichment (hypergeometric distribution) across 139 samples on the 340 diseases. The enrichment significance (adjusted −log_10_*P*-value) for each disease across samples was used to infer cell type and function related associations.

**Figure 2.**
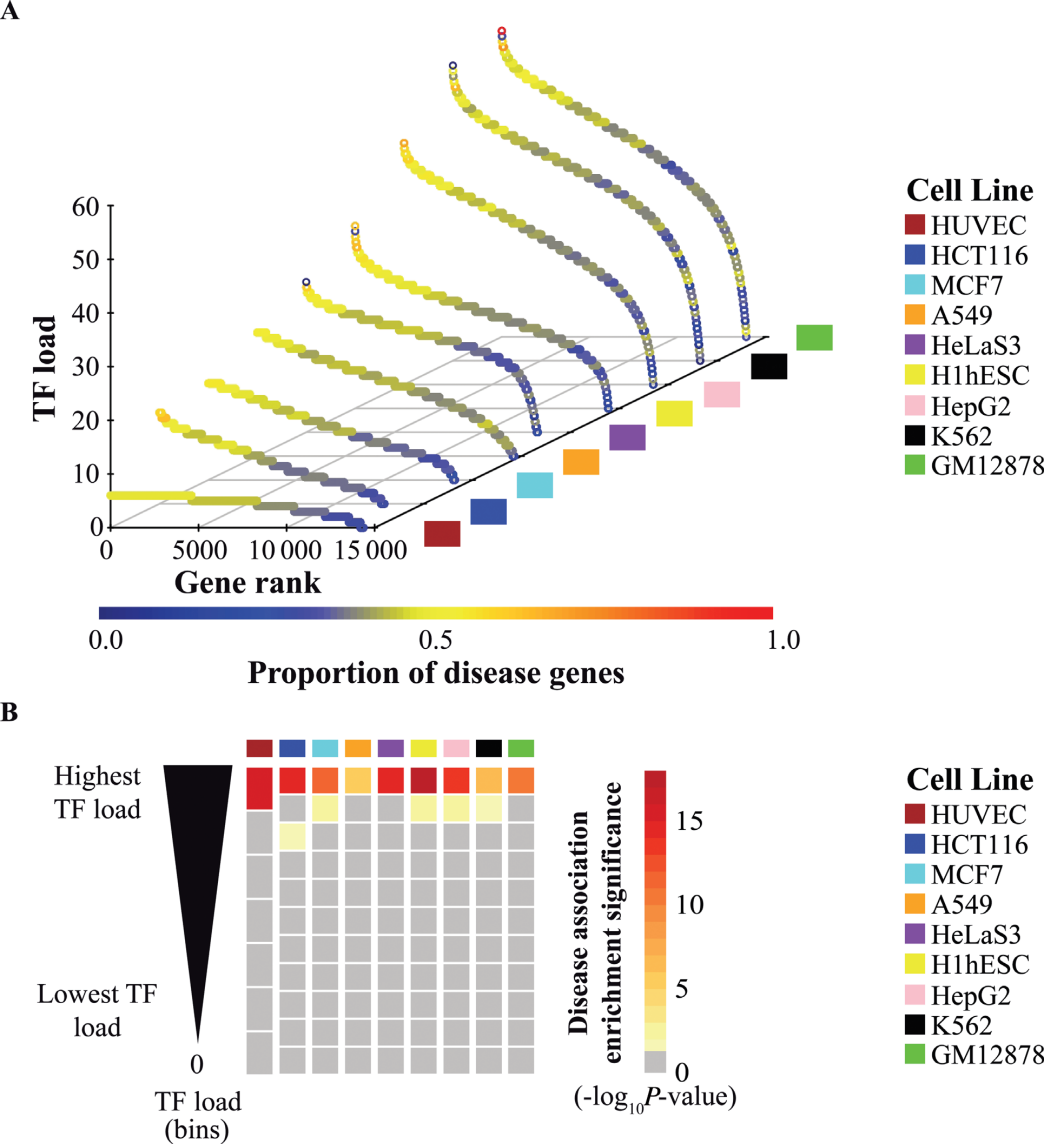
TF load enriches for disease association. Based on ENCODE data from 93 TFs across nine cell lines, the proportion of disease genes is higher among genes with high TF load. (**A**) 3D scatter plot of the TF load per gene and proportion of disease associated genes across nine cell lines. The proportion of disease genes is higher among genes with more TFs. Genes were ranked based on the number of TFs falling within their regulatory region (as defined in Methods). The TF load is depicted on the *z*-axis and the gene rank based on the TF load on the *x*-axis. Data from genes without the H3K4me3 mark within ±1000 bp of the transcription start site are not shown. The nine ENCODE cell lines are shown across the *y*-axis. 6167 disease genes were considered based on the DisGeNET version 2.1 associations (minimum association score of 0.08). The proportion of disease genes among all genes with each unique observed TF load is represented by the color gradient on the TF load for each cell line. (**B**) Heatmap depicting the statistical significance of the enrichment for disease associated genes in all TF load bins (adjusted −log_10_*P*-value), across nine cell lines. For each cell line, genes were grouped based on the number of TFs into deciles, and genes without TFs grouped separately. To avoid different bins having genes with the same TF load, the deciles were adjusted to contain all genes with the same number of TFs as the last gene in the decile. Using the set of 6167 disease associated genes derived from the DisGeNET, hypergeometric tests for each bin were performed. The statistical significance is indicated by the color gradient. Values below 1.301 (i.e. adjusted *P*-values larger than 0.05) are shown in gray and not considered significant. The enrichment significance is highest in the top bin of each cell line.

To investigate whether this is a general finding across different cell types and gene categories, we took advantage of the numerous ChIP-Seq data sets of TF binding produced by the ENCODE project in a number of cell types (19). In detail, we used the existing TF binding data for a total of 93 different previously manually curated TFs (23) from nine ENCODE cell lines, representing different cell and tissue types (Figure 1, Supplementary File 2; see Methods for details). The number of assayed TFs per cell line varied from 6 TFs in HUVEC cells to 65 TFs in GM12878 cells. In addition we used ChIP-Seq data for H3K4me3 from each cell line to identify the putative active genes and associated all TF binding events to their proximal protein-coding genes marked by H3K4me3 following the ‘BasalPlusExtension’ rule of the GREAT tool (17). The number of unique associated TFs per each gene was then calculated and the genes were ranked according to the number of associated TFs, i.e. their regulatory load in each cell type (Figure 2A). The number of associated TFs ranged from 0 TFs per gene to as many as 57 TFs per gene for the genes with highest load in the GM12878 lymphoblastoid cell line (Supplementary File 2). Finally, all genes were classified either as disease-associated or non-disease-associated based on the evidence in the DisGeNET database (using a cut-off score of 0.08 for disease-association to exclude associations based only on text mining) (Figure 1; see methods for details) (4,5).

When focusing on the genes ranked according to their TF load, a similar pattern emerges in each cell line, independent of the number of assayed TFs: the proportion of disease-associated genes is usually close to or above 40% for the genes with highest TF load while for the majority of genes this proportion remains at 10–35% (Figure 2A). To test whether the observed enrichment is statistically significant, we ranked the H3K4me3 marked genes in each cell line into 10 bins of comparable size according to their TF load (6 bins in case of HUVEC cells) with an additional 11th bin in case the gene was not associated with any TF (Figure 2B). Next, the enrichment of disease-associated genes within each bin was tested using the hypergeometric distribution (see Methods for details). As shown in Figure 2B, only the bins of genes with highest TF load show a significant enrichment of 1.301 or higher (adjusted −log_10_ *P*-value corresponding to 0.05) for disease-association with bins based on top 10% genes always showing the most significant enrichment. Importantly, similar results were also obtained when using Gene Set Enrichment Analysis instead of hypergeometric distribution (15).

In conclusion, genes under combinatorial control from multiple TFs are enriched for disease association across multiple cell types, suggesting high regulatory load as a common feature of genes implicated in human diseases.

### High transcription factor load correlates with high abundance of active enhancers

**Figure 3.**
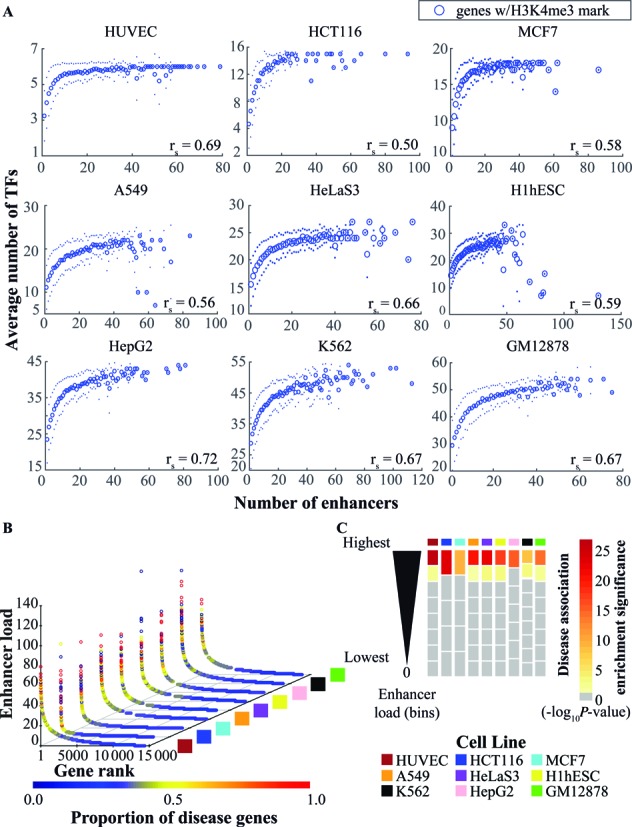
Enhancer load enriches for disease association. (**A**) Plots of the average number of TFs (*y*-axis) for each unique number of enhancer peaks per gene (*x*-axis) for nine cell lines. A positive correlation between the two is observed and the Spearman's rank correlation coefficient (r) is shown on the lower right corner of each plot varying from 0.5 (HCT116) to 0.72 (HepG2). (**B**) 3D scatter plot of the enhancer load per gene and proportion of disease associated genes across nine cell lines. The proportion of disease genes is higher among genes with more enhancers. Genes were ranked based on the number of enhancer peaks falling within their regulatory region (as defined in Methods). The enhancer load is depicted on the *z*-axis and the gene rank based on the enhancer load on the *x*-axis. Data from genes without the H3K4me3 mark within ±1000 bp of the transcription start site are not shown. The nine ENCODE cell lines are shown across the *y*-axis. A set of 6167 disease genes were considered based on the DisGeNET version 2.1 associations (minimum association score of 0.08). For each cell line, the proportion of disease genes among all genes with each unique enhancer load observed was calculated. This proportion is represented by the color gradient on the enhancer load for each cell line. (**C**) Heatmap depicting the statistical significance of the enrichment for disease associated genes on all the different bins of genes based on their enhancer load (adjusted −log_10_*P*-value), across nine cell lines. For each cell line, genes were grouped based on the number of enhancers into deciles, and genes without enhancers grouped separately. To avoid different bins having genes with the same enhancer load, the deciles were adjusted to contain all genes with the same number of enhancers as the last gene in the decile. Using the set of 6167 disease associated genes derived from the DisGeNET, hypergeometric tests for each bin were performed. The statistical significance is indicated by the color gradient. Values below 1.301 (i.e. adjusted *P*-values larger than 0.05) are shown in gray and not considered significant. The enrichment significance is the highest in the top enhancer load bin in all nine cell lines.

While presence of a TF binding event in proximity of a target gene could be indicative of either activation, repression or even no regulation by the TF, the presence of enhancer markers such as H3K27ac are indicative of active enhancers engaged in transcriptional activation via chromatin looping (32,33). To see whether the observed disease-association enrichment of genes under high TF load could be more easily observed by analyzing only few chromatin modifications, we used the H3K27ac ChIP-Seq data for active enhancers produced by the ENCODE project from the corresponding cell lines. Comparison of the average TF load and corresponding number of enhancer peaks at each open gene across the cell lines revealed a clear positive correlation, arguing that most genes under high TF load are also identifiable by a high active enhancer load (Figure 3A). Similar conclusion can be made when the genes are binned in comparable sized groups according to their TF or enhancer loads and analyzed for enrichment of genes within each bin (Supplementary Figure S1, Supplementary File 5). For example, the bins containing genes with highest TF load are significantly enriched for genes with highest enhancer load, and vice versa, the bins of genes with no associated TFs are also enriched for genes with no enhancers.

Based on the obtained correlations, we asked whether ranking of genes according to their enhancer peak abundance would also reveal higher proportion of disease-associated genes among the top ranking genes, similarly to high occupancy by multiple TFs. Indeed, the top ranking genes with highest enhancer load showed higher proportion of disease genes while genes associated with less than 10 enhancer regions rarely show a disease-gene proportion higher than 40% (Figure 3B). Again, the enrichments are also highly significant for the genes under the highest enhancer load in each cell line when tested with the hypergeometric distribution after grouping genes in comparable size bins, with top bins showing the most significant enrichments (Figure 3C). And yet again, similar results were also obtained when using Gene Set Enrichment Analysis instead of hypergeometric distribution (15). Moreover, similar enrichment patterns are also visible when more stringent groups of disease genes (DisGeNET score cut-off 0.2 or genes of monogenic diseases from OMIM database) are used (Supplementary Figure S2). Importantly, the disease-gene proportion profiles obtained using the enhancer load data appear more comparable between the different cell lines than in the TF load analysis that is highly dependent on the number and identity of the assayed TFs.

Taken together, the TF load of accessible (H3K4me3 marked) genes is positively correlated with the number of associated active enhancer peaks and the genes with highest enhancer load are enriched for known disease-relevant genes. This could allow the identification of novel disease genes through ChIP-Seq analysis of enhancer load using histone marks such as H3K27ac.

### Cell type-selective disease-association of genes controlled by multiple active enhancers

H3K4me3 and H3K27ac profiles have already been mapped in numerous different tissue and cell types, allowing us to extend our analysis beyond the nine cell lines from the ENCODE project. To this end, we collected additional pre-processed ChIP-Seq data mapping both modifications from the ENCODE project (19), NIH Epigenomic Roadmap Consortium (21) and BLUEPRINT Epigenome project (20), obtaining a total of 139 sample sets corresponding to 96 different cell types and tissues (Figure 1, Supplementary File 2). For each sample set we performed the enhancer-to-gene association as described in Methods and binned the H3K4me3 marked genes according to their enhancer load to identify the genes under high regulatory load (in the top bin) in each sample set (Supplementary File 3). To compare the top bins, the Jaccard similarity index was calculated for the pair-wise combinations of the 139 samples (Supplementary File 4). Interestingly, the genes with high regulatory load varied a lot between the different cell types and tissues, with most cell types showing lower than 30% similarity when compared with the Jaccard similarity index (Supplementary Figure S3). Consistent with previous reports, the similarity was highest between the cell types from the same tissue, function or developmental origin.

**Figure 4.**
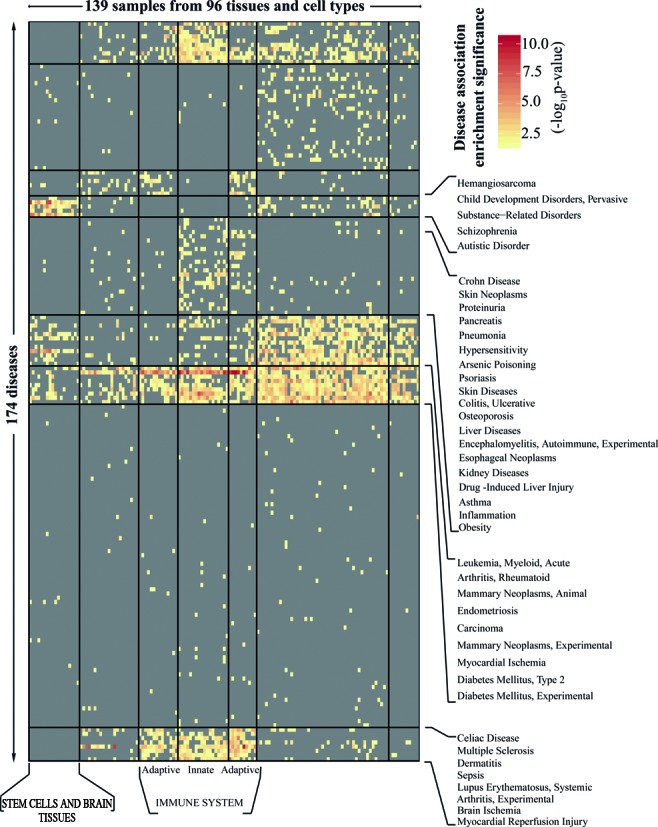
Cell type-selective disease-association of genes under high regulatory load. Heatmap showing the statistical significance (adjusted −log_10_*P*-value) of the disease association enrichment of the high enhancer peak load genes across 139 samples. For each of 139 samples, the set of genes with highest enhancer load (top 10% bin) was taken to perform hypergeometric enrichment tests for disease association on 340 diseases (disease associated genes from DisGeNET version 2.1, minimum 15 genes with a minimum score of 0.08 per disease). The significance of each test is represented as adjusted −log_10_*P*-value for the 139 samples (columns) across 174 diseases (rows), as indicated by the color gradient. Values below 1.301 (i.e. adjusted *P*-values larger than 0.05) are shown in gray and not considered significant. 166 diseases did not have an adjusted −log_10_*P*-value of at least 1.301 in any of the 139 samples. The R package ‘blockcluster’ (24) was used to perform the clustering for samples and diseases resulting in the observed pattern. Supplementary Figure S4 shows the same heatmap with names of all samples and diseases included and Supplementary File 4 contains the details of the diseases and samples as ordered in the heatmap.

Based on this cell-type selectivity of the high regulatory load genes, we hypothesized that high regulatory load would also enrich for diseases in a cell type-selective manner, and possibly allow informative links between different diseases and cell types. To test this, we collected all 340 diseases from DisGeNET database that had at least 15 associated genes with a minimum score of 0.08 (Supplementary File 1). Next, the enrichment of genes associated to each of these diseases was tested separately in all 139 sets of high regulatory load genes derived above based on the number of associated H3K27ac peaks (Supplementary File 3) to obtain a matrix of cell type- and disease-selective significant enrichments (Figure 4, Supplementary Figure S4, Supplementary File 4). A total of 174 diseases showed significant enrichment (adjusted −log_10_ *P*-value ≥ 1.301) in the high regulatory load genes of at least one cell type. Figure 4 shows bi-clustering of the diseases and cell types or tissues according to the enrichment profiles. As expected, cell types are clustered together largely according to their function or developmental origin. For the different diseases the clustering patterns are not as obvious but still interesting clusters emerge. The largest cluster (second from the bottom) consists of 76 various diseases that are fairly weakly enriched in only one or a few different cell types or tissues. On the contrary, only very few diseases (mostly in the third disease cluster from bottom) showed enrichment in almost all cell types. These include many systemic diseases or syndromes such as type 2 diabetes and rheumatoid arthritis or broad categories related to cancer such as carcinoma and leukemia. Among the different cell types, the enrichments in the high regulatory load genes of the immune cells included many different diseases. Diseases like multiple sclerosis and systemic lupus erythematosus were particularly enriched for cells of both innate and adaptive immune systems while other autoimmune and inflammatory diseases, including Crohn's disease and asthma as well as acute inflammations, induced for example by pneumonia and drug-induced liver injuries, were preferably enriched in high regulatory load genes of the innate immune cells. Finally, the most selective disease enrichments were observed for the high regulatory load genes of the different brain regions and the closely clustering pluripotent stem cells. Most of these showed enrichments mainly for the disease groups such as pervasive child development disorders, substance-related disorders, schizophrenia and autistic disorder. Finally, among the cell types with a particularly low number of disease-associations, pancreatic islet was associated to only seven different diseases, with the most significant disease-association to type 2 diabetes. Such selective disease associations might reflect the highly specialized functions of the cell types like islet cells and stem cells, but might also reflect the fact that relatively little is still known about the disease mechanisms in tissues like brain.

In summary, the genes under high regulatory load vary between different cell and tissue types and, consistently, are enriched for different diseases in different cell types, often in accordance with known involvement of those cell types in the respective diseases. Therefore, identification of genes under high regulatory load using epigenomic data for active enhancers could guide identification of novel disease-associated genes in a cell-type-selective manner.

### Identification of novel putative disease genes in human monocytes

Among the 139 samples of enhancer data the cell type with most samples are the monocytes that are innate immune cells involved in a wide range of diseases. To test the prediction of novel disease genes based on their regulatory load, we combined the high regulatory load genes from 10 monocyte samples to obtain an extensive list of 3131 monocyte high regulatory load genes. Next we compared this list to high regulatory load genes in all other samples in order to obtain a unique list of 82 monocyte-specific high regulatory load genes (Supplementary File 6). From these genes 25 were already included as disease-associated genes in DisGeNET version 2.1 (from 5th of May 2014) used in our analysis above, and 15 of them were associated to diseases with known involvement of monocytes or cell types derived from them (e.g. arthritis, pycnodysostosis, myeloid leukemia and properdin deficiency). This leaves 57 monocyte-specific high regulatory load genes that we expect to have higher probability of being associated with disease, especially in monocytes (Supplementary File 6).

After the initial submission of the manuscript a new version of DisGeNET (version 3.0, May 2015) was released, including 767 novel high confidence disease genes (cut-off score of 0.2 including only strong evidence associations), 710 of which are included in the gene background set used for our epigenomic analysis (Supplementary File 1). Searching for the 57 predicted monocyte disease genes described above among the 710 newly associated disease genes showed that as many as 14 of them had now been included as high confidence disease genes during the year between the two releases. These include genes such as *NUSAP1* and *MS4A6A* that are associated to glomerulonephritis, an IgA nephropathy (34); *GPBAR1* that is highly expressed in intestinal monocytes of patients with inflamed Crohn's disease (35), and; *SYNJ1* and *PLD3* that are both associated to Parkinson's disease and Alzheimer's disease (36–39). While the latter two genes have been studied mainly in the context of neurons, both associated neurodegenerative diseases have also a well-established neuroinflammatory component. And interestingly, *PLD3* shows the highest expression across all cell types in monocytes and related cell types, similarly to another non-classical phospholipase D family member, *PLD4*, that is known to be involved in microglial phagocytosis in the brain (40,41).

Finally, to perform a more robust test of the prediction power of high regulatory load for disease-gene association, we tested whether more of the newly associated 710 disease genes from DiGeNET version 3.0 could be found among the high regulatory load genes across all 139 samples used in our analysis. Notably, 469 or 66% of the new disease genes could indeed be found among the high regulatory load genes across the analyzed cell types, a significantly higher fraction than expected by chance (hypergeometric test, *P*-value = 1.6880e-12). Thus, arguing that high regulatory can guide identification of novel disease-associated genes.

### Comparison of high regulatory load and super-enhancer genes

**Figure 5.**
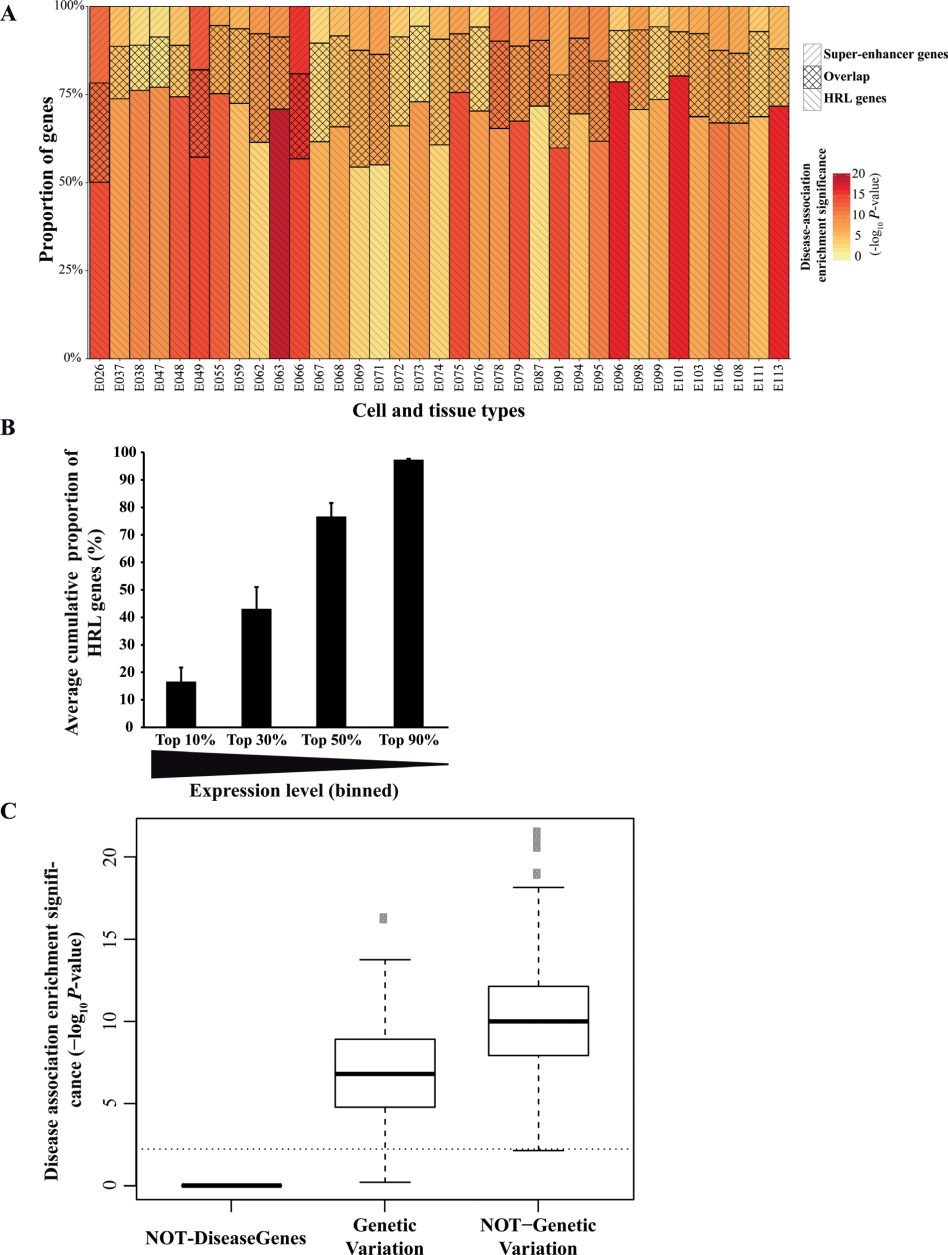
Features of the disease association of high regulatory load genes and comparison to super-enhancer genes. (**A**) Proportions of high regulatory load genes (descending diagonal stripes), super-enhancer genes (asscending diagonal stripes) and their overlap (crossing diagonal stripes) from their combined count in 35 samples from Epigenomics Roadmap consortium are indicated as cumulative bars. Code name for the each sample corresponds to those described in (21) and can be found in Supplementary File 2. Heatmap shows the statistical significance (adjusted −log_10_*P*-value) of the disease association enrichment of either the super-enhancer genes (upper part of the bar) or high regulatory load genes without super-enhancer genes (lower part of the bar). (**B**) Average cumulative proportion (±SD) of high regulatory load genes within the top 10%, top 30%, top 50% and top 90% of highest expressed genes across 38 RNA-Seq samples from Roadmap Epigenomics consortium corresponding to samples detailed in Supplementary Files 2 and 7 (see Methods for details). (**C**) Boxplot showing the statistical significance (adjusted −log_10_*P*-value) of the enrichment for disease association of the top enhancer load bin from each sample (n = 139) obtained considering the 2832 genes for which the association to a disease is defined as ‘genetic variation’ (based on the DisGeNET) *versus* the 4596 genes for which the association type is other than ‘genetic variation’. Adjusted −log_10_*P*-value values below 1.301 (gray dashed line), i.e. *P*-values larger than 0.05 are considered non-significant. While disease-associated genes based on genetic variation enrich on the top enhancer load bin, this enrichment is not lost when excluding those genes and keeping disease-associated genes based on other types of association evidence based on DisGeNET (‘altered expression’, ‘biomarker’, ‘post-translational modification’ and ‘therapeutic’). The set of 13 071 genes in our background set that are not disease associated was used as a control, showing no significant enrichment of non-disease genes among the genes with more enhancer peaks across all 139 samples.

High regulatory load from multiple active enhancer peaks is conceptually very similar to previously described super-enhancers or stretch-enhancers that have also been associated to disease through high occurrence of disease-associated genetic variants within them (10,11). To compare high regulatory load genes with super-enhancer genes we used the 35 Epigenomics Roadmap samples for which mapped reads of H3K27ac ChIP-Seq data were available to call super-enhancer peaks in those samples (see Methods for details). This yielded between 300 and 900 super-enhancer genes per sample. Overlapping these genes with previously identified high regulatory load genes from the same samples showed that in all cases the majority (on average 67.9%) of the super-enhancer genes belong also to the group of high regulatory load genes (Figure 5A). However, these make up only 13–37% of all high regulatory load genes.

As expected, also super-enhancer genes were enriched for disease-association in all tested cell types (Figure 5A). This led us to wonder if the observed disease-associations for high regulatory load genes are simply due to the included super-enhancer genes. To address this possibility we generated separate lists of high regulatory load genes that exclude super-enhancer genes in all 35 samples and tested these genes for their disease-association enrichment. Importantly, in each case the remaining high regulatory load genes enriched for disease-association also when the super-enhancer genes were excluded from the analysis (Figure 5A).

Given that high regulatory load genes are associated with high number of active enhancers it could be assumed that they are also higher expressed than other genes on average. Consistently, this has already been shown to be the case for super-enhancer genes (9). To test this for high regulatory load genes we obtained normalized RNA-seq data for 38 cell types and tissues for which they were available from the Epigenomics Roadmap consortium. In keeping with the hypothesis, the high regulatory load genes showed approximately 2.1-fold higher expression levels than all genes on average (Supplementary File 7). And looking at all known disease genes, they too exhibited approximately 1.65-fold higher expression levels. This was mainly based on the two largest disease categories called ‘Biomarkers’ and ‘Genetic Variation’ which both showed the same average expression levels while the other smaller categories all showed even further elevated levels of expression between 2.65- to 3.2-fold above the average of all genes.

Based on these results we asked whether the high regulatory load genes could be obtained simply by focusing on the highest expressed genes in each cell type. To do this we grouped the genes in each sample according to their expression depending whether they were in the top 10%, top 30% or top 50% of highest expressed genes or in top 90% group containing most genes. Next we asked how large proportion of the high regulatory load genes in each cell type could be found in each group. As shown in Figure 5B, on average across the cell types, only 16.6% of high regulatory load genes could be found among the comparably sized top 10% of highest expressed genes. And only when considering the higher expressed half of all genes (top 50%) could 76.7% majority of high regulatory load genes be obtained.

Taken together, the majority of super-enhancer genes can be identified among the genes with high regulatory load, but they do not alone explain the observed disease-association enrichment of high regulatory load genes. Similarly to super-enhancer genes, both high regulatory load and disease genes show above average expression levels but expression level alone serves as a poor predictor of high regulatory load.

### High regulatory load genes are not associated to disease only by genetic variation

As much as 90% of disease-associated genetic variants are located outside of coding genic sequences in humans and recent work integrating epigenomic analysis with GWAS has showed that around 60% of the variants are coinciding with active enhancers (6–8,19). This is particularly true for super-enhancers that serve as binding platforms for combinations of multitude of TFs (10,11). While the observed enrichment of disease genes among the genes under high regulatory load is not only due to super-enhancer genes, it might still be due to increased likelihood of these genes being associated to genetic variants.

In order to assess whether this is sufficient to explain our findings, we divided all protein coding genes into three categories: (i) genes not associated to any disease with a score above 0.08 according to DisGeNET database (13 071 genes); (ii) genes associated to diseases based on evidence for genetic variation (score ≥ 0.08; 2832 genes), and; (iii) genes associated to diseases based on other evidence than genetic variation (score ≥ 0.08; 4596 genes). Subsequently, enrichment of each of these gene sets in the high regulatory load genes of all 139 samples was tested and the boxplots of the adjusted enrichment *P*-values are depicted in Figure 5C. Importantly, the genes not associated to any disease also did not show any enrichment in any of the samples while genes associated to diseases through genetic variation showed significant enrichment in all samples with a median adjusted −log_10_ *P*-value of 6.1. However, also the other disease-associated genes, without evidence for genetic variation, showed a highly significant enrichment among all 139 sets of high regulatory load genes with a median adjusted −log_10_ *P*-value of 9.0. Thus, suggesting that there could be also other explanations for the frequent disease-association of the high regulatory load genes besides their higher likelihood of being affected by a genetic variation.

### High regulatory load genes are involved in multiple pathways

**Figure 6.**
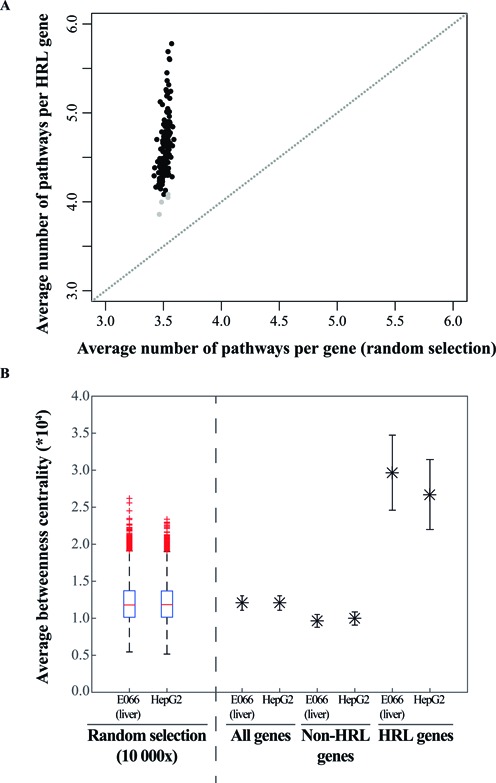
High regulatory load genes appear on average in more pathways and exhibit higher betweenness centrality than randomly observed. (**A**) Plot of the average number of pathways per gene for the high regulatory load genes across 139 samples (one dot per sample) as a function of the average number of pathways per randomly selected equal number of genes. While randomly selected genes appear on average in 3.52 pathways (constant number of pathways, the variation over the *x*-axis is very low), high regulatory load genes appear on average in 4.66 pathways and present a much higher variation (min. 3.86, max. 5.78), suggesting their importance as network nodes. 282 KEGG pathways were considered. For each set of highest enhancer load genes from the 139 samples, the average number of KEGG pathways they belong to was calculated (*y*-axis). Equal numbers of randomly selected genes were taken in a 10 000 fold re-sampling and the average number of pathways they belonged to are depicted on the *x*-axis. The statistical significance was determined by a re-sampling test and the significant (*P* ≤ 0.05) and non-significant samples are shown as black and gray dots, respectively. (**B**) Betweenness centrality of genes under high regulatory load in liver disease gene network (for illustration see Supplementary Figure S5). A liver disease gene network was constructed as described in Methods and the betweenness centrality was calculated for each gene present in the network and potentially expressed in either liver sample based on the H3K4me3 mark. Boxplots (left side of the dashed line) represent the distribution of average betweenness centralities for 10 000 sets of equal numbers of randomly selected network genes. Asterisks (right side of the dashed line) represent the average betweenness centralities (±SEM) of all genes, all genes except those under HRL in primary liver tissue (E066) or HepG2 cell line and, high regulatory load genes in the two different samples as indicated. The average betweenness centrality of high regulatory load genes is significantly higher than for other genes as determined by a re-sampling test.

The positioning of disease genes as central hubs in gene-regulatory or protein-protein interaction networks has been suggested to make the genes more likely to cause or be affected by perturbations than what would be the case for more peripheral genes (42). Indeed, one of the putative explanations for the higher occurrence of disease association among the genes under high regulatory load could lie within their role as central network nodes and as integration points within and between pathways. To see whether this hypothesis is supported by the current pathway knowledge we obtained the node information for all KEGG pathways (27) and calculated in how many pathways the high regulatory load genes occur on average in each of the 139 samples (Figure 6A). This was compared to the average pathway occurrence of an equal number of randomly selected H3K4me3 marked genes from each sample. Interestingly, in 135 of the 139 samples the average pathway occurrence was significantly higher (4.66 pathways per HRL gene on average) than for the randomly selected genes (3.52 pathways per gene on average) based on a re-sampling test (see Methods for details) with a large variation up to almost 6 pathways per gene in some cell types. Consequently, the high regulatory load genes occur in more known pathways than other genes on average, suggesting that the identified disease-association enrichment could be due to central role of these genes within biological networks.

### Genes under high regulatory load in liver exhibit high betweenness centrality in liver disease gene network

To directly address the positioning of high regulatory load genes in biological and disease networks, we constructed a liver disease-specific network covering 137 liver diseases that comprises of 3775 genes (nodes) and 8278 interactions (edges) based on human protein interactions from the Human Protein Reference Database (HPRD) (29) (see Methods for details of the network construction). An illustration of the network with positioning of high regulatory load genes can be found in Supplementary Figure S5. Next we obtained the lists of all H3K4me3 marked and high regulatory load genes in two liver samples, primary liver tissue and HepG2 hepatocarcinoma cell line, that were also present in the newly constructed liver disease gene network. As additional control, we created 10 000 lists of random selection of genes of equal numbers from both samples. Finally, to analyze the positioning of the high regulatory load genes we calculated the betweenness centrality for each gene in the network and compared the average betweenness centralities of the high regulatory load genes to the different control gene lists. Notably, while randomly selected genes showed similar mean betweenness as all genes, the high regulatory load genes showed in both samples almost 3 times higher betweenness than either of these control groups (Figure 6B). This is consistent with the somewhat lower betweenness centrality of the gene group where high regulatory load genes have been excluded. Accordingly, high regulatory load genes occupy the more central nodes within the liver disease gene network.

### Genes under high regulatory load at transcriptional level have longer 3′ UTRs and contain more miRNA binding sites

**Figure 7.**
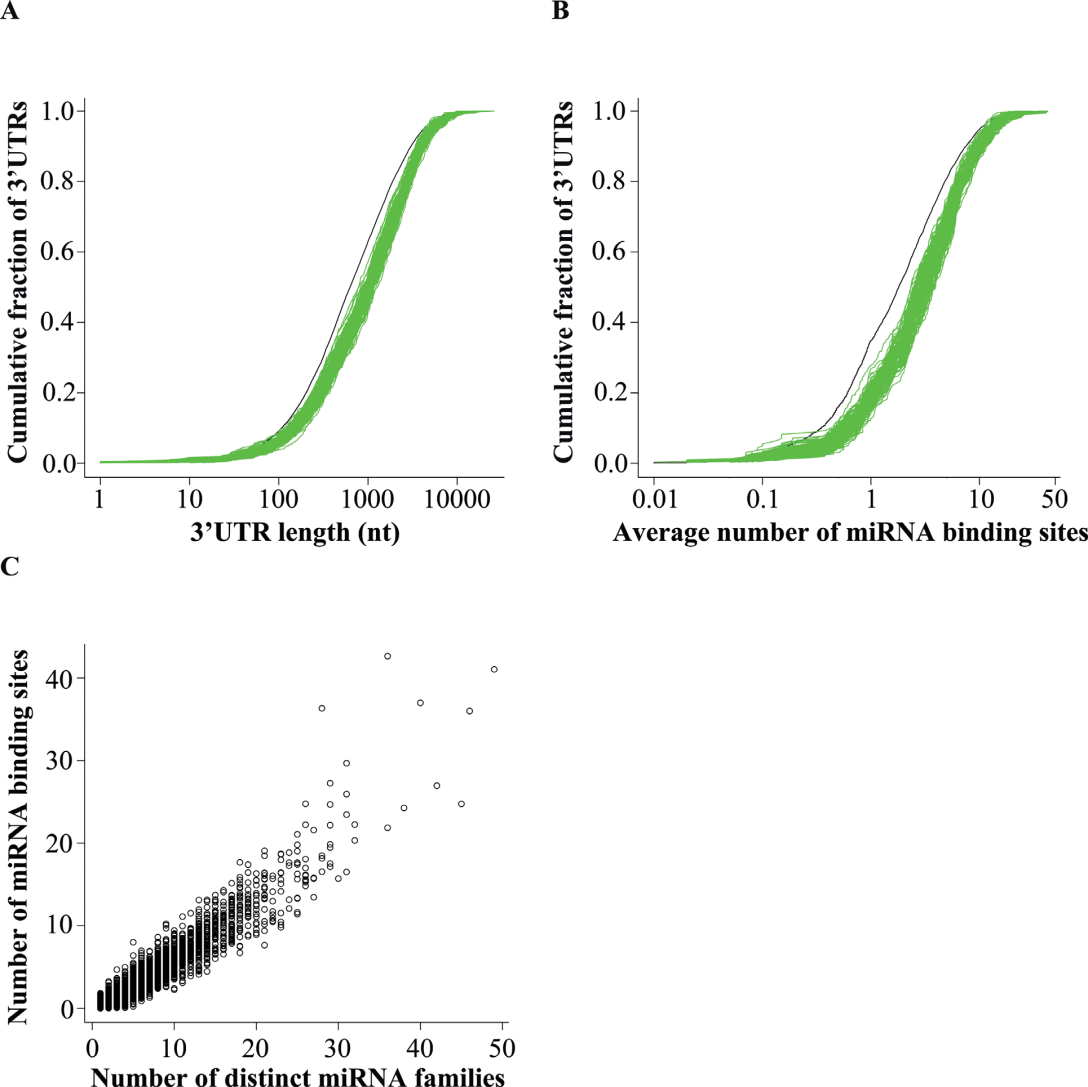
Genes under high regulatory load at transcriptional level have longer 3′ UTRs. (**A**) Distributions of 3′ UTR lengths in 139 sets of high enhancer load genes from different samples (each depicted by a green line) and in a background set of 16 307 3′ UTRs (depicted by the black line). The average 3′ UTR length of all mean lengths of the high enhancer load genes was 1695 nt, 39% longer than the average length of 1213 nt for the background set genes. For 138 samples the length was significantly longer than for the background set (Kolmogorov-Smirnov test, see methods). (**B**) Distributions of counts of predicted conserved miRNA binding sites (TargetScan 6.2) in 139 sets of high enhancer load gene 3′ UTRs from different samples (each depicted by a green line) and in a background set of 16 307 3′ UTRs (depicted by the black line). (**C**) The total number of predicted miRNA binding sites per 3′ UTR (*y*-axis) is positively correlated with the number of distinct miRNA families targeting the 3′ UTR (*x*-axis) across all genes.

Since high regulatory load genes appear to function as important nodes in biological pathways and integrate multiple signals at the transcriptional regulation level, we asked whether a similar finding could be made also at the other regulatory levels. More specifically, we assumed that high enhancer load genes might be under higher regulatory load also at the post-transcriptional level. Post-transcriptional regulation of mRNA stability and translation takes place mainly via the binding of miRNAs and various RNA-binding proteins to their regulatory regions in the mRNAs 3′ UTR with longer 3′ UTRs allowing higher number of regulatory regions (43,44). To test whether the 3′ UTRs of genes under high regulatory load from multiple enhancers in different cell types could in principle occupy more regulatory regions than all genes on average, we collected the 3′ UTR lengths for all genes and compared the 3′ UTR lengths in the different gene sets (Figure 7A, see Methods for details). Curiously, in 138 of the 139 samples the 3′ UTR length distribution was significantly longer for the top bin of highest enhancer load genes than for all genes (Kolmogorov-Smirnov test). The average 3′ UTR length for all genes was 1213 nt while mean of all high regulatory load genes means was 1695 nt, i.e. 482 nt (or 39%) longer. To further see whether these longer 3′ UTRs indeed contain more regulatory regions, we analyzed the distribution of conserved miRNA binding sites predicted by the TargetScan software (31) within the 3′ UTRs (Figure 7B). In keeping with the longer length, the 3′ UTRs of the high enhancer load genes from each of the 139 sample sets contain significantly more miRNA binding sites than other genes on average, making them more prone to post-transcriptional regulation. In general, across all genes, the increased number of miRNA binding sites strongly correlates with the number of miRNAs from distinct miRNA families (Figure 7C), suggesting that the observed high number of miRNA binding sites also reflects targeting by multiple different miRNA families. Thus, the high regulatory load genes appear to be under combinatorial regulation by distinct regulators mediating multiple signals both at transcriptional and post-transcriptional level.

## DISCUSSION

Much of the research is focused on elucidating the roles of selected few genes in human health and disease although this emphasis is not warranted by their connectivity, conservation or other features when compared to the less studied genes (1). The advent of different genome-wide approaches has allowed an improved ‘equality’ among genes and unbiased approaches to prioritize the previously uncharacterized genes based on these vast data sets will be increasingly important. Here we show that genes regulated by a high TF load are more likely to be disease-associated genes and can be identified across cell types through epigenomic mapping of active enhancers. The sets of high regulatory load genes vary between cell types, thereby allowing identification of putative disease-associated genes in a cell type-selective manner. Disease-association of these genes appears to rely on multiple different categories of association evidence and we propose central role within biological networks as one of the likely explanations for the observed enrichment. In keeping with the putative role as integrators of multiple signals between pathways, the high regulatory load genes appear also to be targeted by more post-transcriptional regulators such as miRNAs. This is consistent with earlier findings for positive correlation between numbers of TF and miRNA binding sites (45), and provides an additional feature that could be shared by the most relevant genes.

High load of active enhancers often assumes high expression levels of the target genes, a concept already suggested by many studies (9,10; Supplementary File 7). Therefore it is somewhat paradoxical why these genes would also be targeted by higher number of post-transcriptional regulators, such as miRNAs, that are mainly repressing their target genes. One possibility is that the miRNA regulation serves as a buffer to keep the abundant expression of the target genes within certain threshold in a robust manner (46). On the other hand, it is known that miRNAs and their target mRNAs are expressed in a mutually exclusive manner, suggesting that the high regulatory load genes could be under strong miRNA-mediated repression in other cell types where they are not occupied by high enhancer load, thus further enforcing their selective expression profiles (47). Consistently, multiple different miRNA binding sites might be needed to allow the repression of the genes by different miRNAs in different cell types.

While analyzing the 3′ UTR lengths we observed that also the coding sequences (CDS) of the high regulatory load genes are longer than the mean of all genes, albeit with smaller (24%) and less significant increase (Supplementary Figure S6A). And importantly, the unspliced primary transcripts are as much as 94% longer (Supplementary Figure S6B). This raises the possibility that these are simply longer genes occupying larger genomic regions, with the higher regulatory association at transcriptional level stemming from this feature. However, the 3′ UTR and CDS lengths of the different genes show no correlation and similar results for 3′ UTR lengths can be obtained when focusing only on enhancers or TF binding sites located upstream of the target genes (Supplementary Figure S6C and data not shown). Therefore, the longer 3′ UTR and indeed an overall longer gene length appear to be inherent features of the high regulatory load genes. This is particularly interesting in the light of the recent observation that human orthologs of mouse essential genes are significantly longer than all other genes on average (48). Indeed, 77% of the 2472 known essential genes with human orthologs are also identified as high regulatory load genes in our analysis and significantly enriched in the top regulatory load bins across all 139 samples (data not shown).

Our data suggest that epigenomic mapping of active enhancers could be used to predict disease-associated genes and thereby prioritize the analysis of previously unknown genes. Current analysis presented in Figure 4 provides an interesting starting point. More detailed analysis of the individual cell types and associated disease enrichments might provide novel insights into relationship of cell types and diseases in question, and in particular, how do the previously unassociated high regulatory load genes within different cell types fit into the network of the already known disease genes. To take the first step we already performed an analysis to identify monocyte-specific high regulatory load genes that could be novel disease genes and show this to be the case for 14 of them. Moreover, genes like *PLD3*, that has been linked to neurodegenerative diseases and studied in the context of neurons, is identified as monocyte-specific high regulatory load gene in our analysis. This suggests that PLD3's association to neurodegenerative diseases might be related to neuroinflammatory component of these diseases, similarly as has been shown for many Alzheimer's disease-associated genetic variants that are enriched in enhancer regions active in inflammatory cells (49).

On the other hand, the enrichment of disease genes associated to many systemic diseases across the high regulatory load genes of most cell types further highlights the need to find interventions to these diseases at whole-body level. Moreover, obtaining epigenomic data from diseased cell types or cells responding to different external signals could provide further interesting target genes for future analysis. In particular, the profiling of previously uncharacterized disease related cell types such as dopaminergic neurons in context of Parkinson's disease could reveal entirely new insights into the underlying epigenetic mechanisms of the disease development (50).

Our comparison of high regulatory load and super-enhancer genes (Figure 5) suggests these features to be two sides of the same coin and a less exclusive definition of these key genes might be beneficial for future analysis. The high enhancer load of the selected genes is largely a reflection of binding of multiple TFs in the regulatory regions of these genes as indicated by the correlations in Figure 2A. Similarly, Joshi has found TF hotspots to be enriched for enhancers and consistently, Siersbak *et al*. have shown super-enhancers to be enriched for TF hotspots (12,51). These findings together with the increased occurrence of these high regulatory load genes in more pathways than expected by chance and with increased betweenness centrality within liver disease gene network (Figure 6) lead us to propose the central role of these genes in regulatory networks as a possible explanation for their increased likelihood for disease association. The high regulatory load genes appear to serve as integration points within and between pathways, possibly also at the post-transcriptional level (Figure 7). Indeed, recent work by Hnisz *et al*. showed embryonic stem cell super-enhancers to consist from several constituents that together serve as binding platforms for a number of TFs to merge signals from multiple signaling pathways (52).

In conclusion, the central role of high regulatory load genes as signal integrators comes with an inherent feature of high enhancer load that can be taken advantage of to identify the genes through epigenomic profiling in a cell type-selective manner. In the future, an integrative approach using high regulatory load together with other features such as network centrality, post-transcriptional regulation, and expression data could be used to prioritize the previously unstudied genes in terms of their relevance for disease.

## Supplementary Material

SUPPLEMENTARY DATA
