# Supplementary material for: Cell type-selective disease-association of genes under high regulatory load
Source: Nucleic Acids Res. 2015 Oct 10;43(18):8839–55. doi: 10.1093/nar/gkv863 (PMC4605313; doi:10.1093/nar/gkv863)
Supplement: SUPPLEMENTARY DATA [file supp_43_18_8839__index.html]

Cell type-selective disease-association of genes under high regulatory load — SUPPLEMENTARY DATA 

# Cell type-selective disease-association of genes under high regulatory load

## SUPPLEMENTARY DATA

- SUPPLEMENTARY DATA
- SUPPLEMENTARY DATA
- SUPPLEMENTARY DATA
- SUPPLEMENTARY DATA
- SUPPLEMENTARY DATA
- SUPPLEMENTARY DATA
- SUPPLEMENTARY DATA
- SUPPLEMENTARY DATA
