# Supplementary material for: Cell type-selective disease-association of genes under high regulatory load
Source: Nucleic Acids Res. 2015 Oct 10;43(18):8839–55. doi: 10.1093/nar/gkv863 (PMC4605313; doi:10.1093/nar/gkv863)
Supplement: SUPPLEMENTARY DATA [file supp_gkv863_nar-01077-z-2015-File009.pdf]

Cell type-selective disease-association of genes under high regulatory load

Mafalda Galhardo, Philipp Berninger, Thanh-Phuong Nguyen, Thomas Sauter and Lasse Sinkkonen

Supplementary Information

Supplementary Figures

Supplementary Figure S1: TF load enriches similar bins of enhancer load. Heatmaps of the hypergeometric distribution enrichment significance (adjusted -$\log_{10}$p-value) of genes binned by TF load (y-axis) across different bins based on enhancer load (x-axis) for 9 ENCODE cell lines (from top to bottom, left to right: HUVEC, HCT116, MCF7, A549, HeLaS3, H1hESC, HepG2, K562 and GM12878). Genes were sorted by regulatory load and grouped in bins (y- and x-axis). Bottom or left side bins contain genes with lower regulatory load than top or right side bins. Bins denoted with "1" contain genes with no associated TF or enhancer. The enrichment significance (adjusted -$\log_{10}$p-value) is depicted by the color gradient, increasing from yellow to red (values ≥ 50 appear in red). Dark grey represents (adjusted -$\log_{10}$p-value) < 1.3, not considered significant. The significance is evident along the diagonal for all 9 cell lines, denoting the concerted increase between TF and enhancer load, with genes in bins of low TF load enriching the highest for genes in bins of low enhancer load and vice-versa, genes of high TF load enriching the most for high enhancer load genes. Supplementary File 5 contains tables with the enrichment significance (adjusted -$\log_{10}$p-value) obtained and the exact TF load per bin (vertically) or enhancer load per bin (horizontally) for each of 9 cell lines.

Supplementary Figure S2: High enhancer load genes enrich for disease association also with more stringent disease gene groups. Heatmaps of the hypergeometric distribution enrichment significance (adjusted -$\log_{10}$p-value) of genes binned by enhancer load across 139 samples. Left side bins contain genes with lower enhancer load than bins on the right side. The

enrichment significance (adjusted -log_{10}p-value) is depicted by the color gradient, increasing from yellow to red. Grey represents (adjusted -log_{10}p-value) < 1.3 (equivalent to p-value > 0.05), not considered significant. The significance is evident on the bins of highest enhancer load on the right side, with orange and red colours. (A) Results using the set of curated disease genes from DisGeNET version 2, minimum association score of 0.2 (7110 genes of which 5853 were in the background set of 19238 protein coding genes). (B) Results using the set of disease genes from the OMIM database, as of June 2015 (4557 genes of which 3483 were in the background set of 19238 protein coding genes).

Supplementary Figure S3: Genes with highest enhancer load vary across 139 samples. Heatmap of the Jaccard similarity index for the pair-wise comparison of genes in the top enhancer load bin across 139 samples. The heatmap is mirrored along the diagonal. Blue denotes few common while red denotes many common genes on the two sets of highest enhancer load genes from any two samples. The predominance of the blue colour reflects an overall low similarity between the genes with highest enhancer load across samples (average similarity lower than 30%). The bottom and right-side color bars denote groups of samples with the same tissue of origin, color-coded on the bottom.

Supplementary Figure S4: Cell type-selective disease-association of genes under high regulatory load. Heatmap from Figure 4 showing the statistical significance (adjusted -log10 p-value) of the disease association enrichment of the high enhancer load genes across 139 samples

and 174 diseases with names of the diseases and samples written out for each case. For more details, see Supplementary File 4.

Supplementary Figure S5: Liver disease gene network. Illustration of the reconstructed liver disease gene network containing 3,775 genes and 8,278 interactions. Red nodes represent the high regulatory load genes from the two liver samples (primary liver (E066) and HepG2) and grey nodes the other liver disease genes and their first neighbours in the network. A higher intensity of red color is observed on the central area of the network, reflecting the higher betweenness centrality of the high regulatory load genes as described in Figure 6.

Supplementary Figure S6: HRL genes have longer CDS and transcripts on average. (A) Distributions of CDS lengths in 139 sets of high enhancer load genes from different samples (each depicted by a green line) and in a background set of 16307 CDSs (depicted by the black line). The average CDS length of all mean lengths of the high enhancer load genes was 1816 nt, 24% longer than the average of length of 1455 nt for the background set genes. (B) Distributions of unspliced transcript lengths in 139 sets of high enhancer load genes from different samples (each depicted by a green line) and in a background set of 16307 unspliced transcripts (depicted by the black line). The average unspliced transcript length of all mean lengths of the high enhancer load genes was 105452 nt, 94% longer than the average length of 54451 nt for the background set genes. (C) The CDS lengths (y-axis) the 3'UTR lengths (x-axis) of the transcripts do not show correlation.

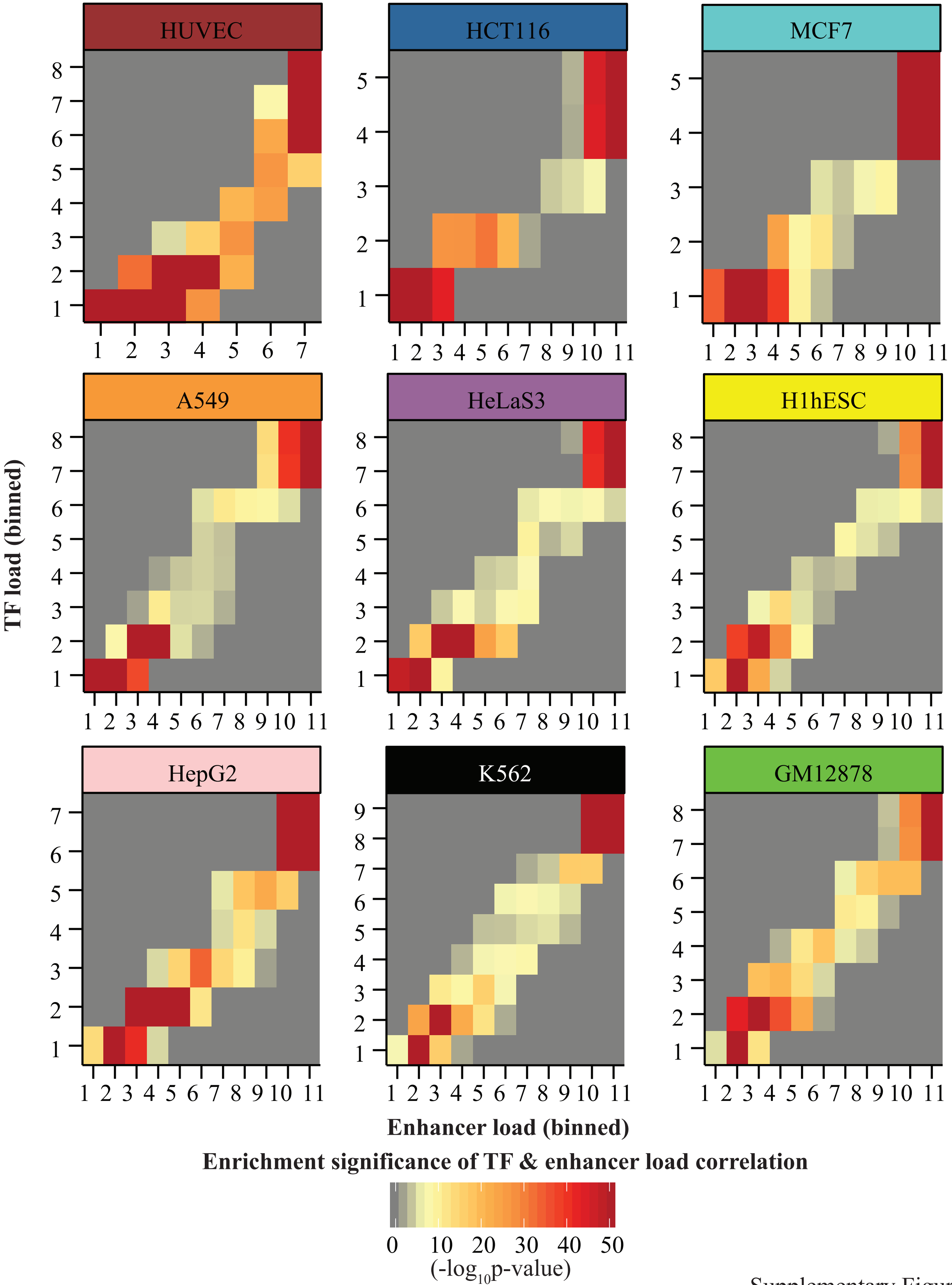

Supplementary Figure S1

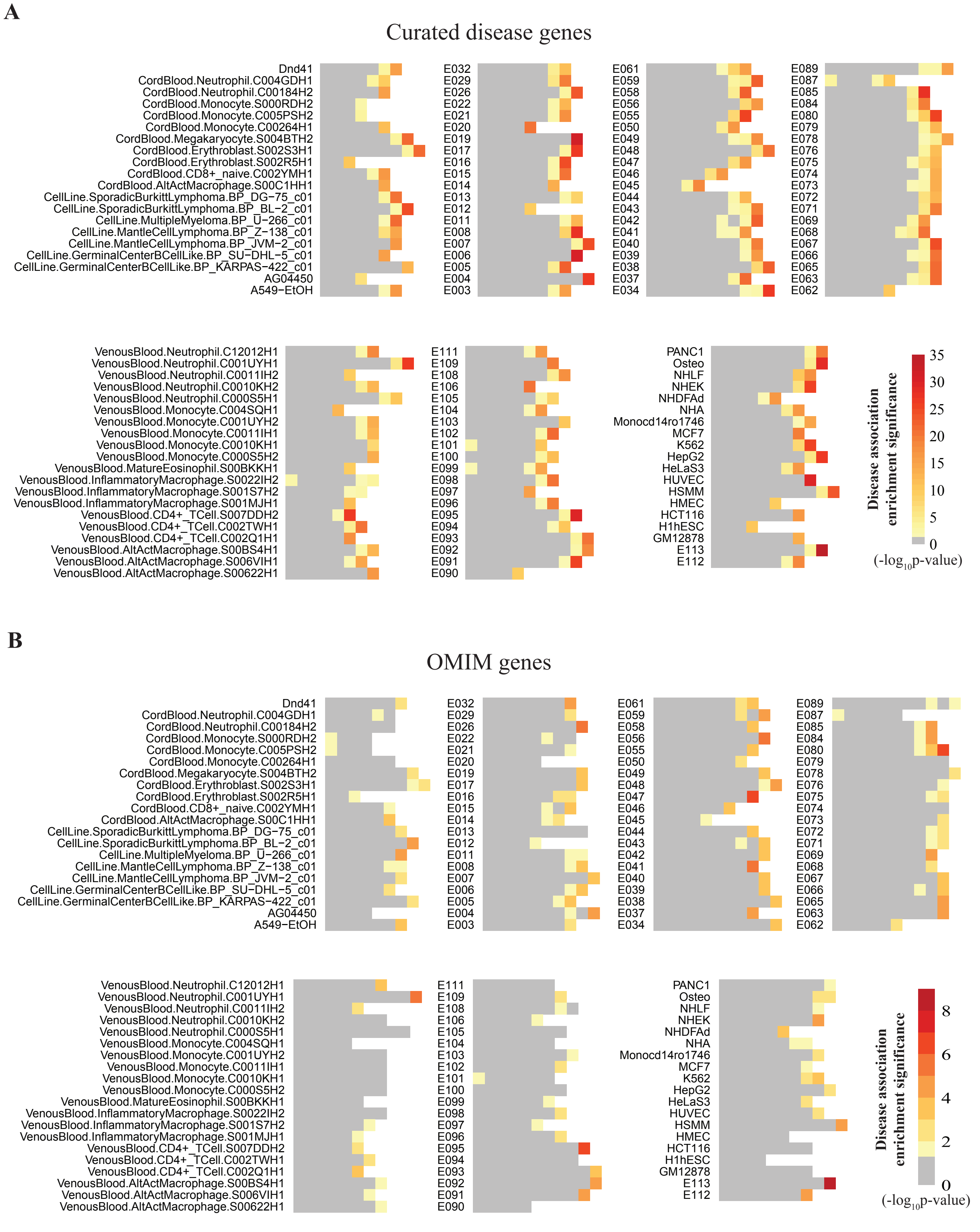

Supplementary Figure S2

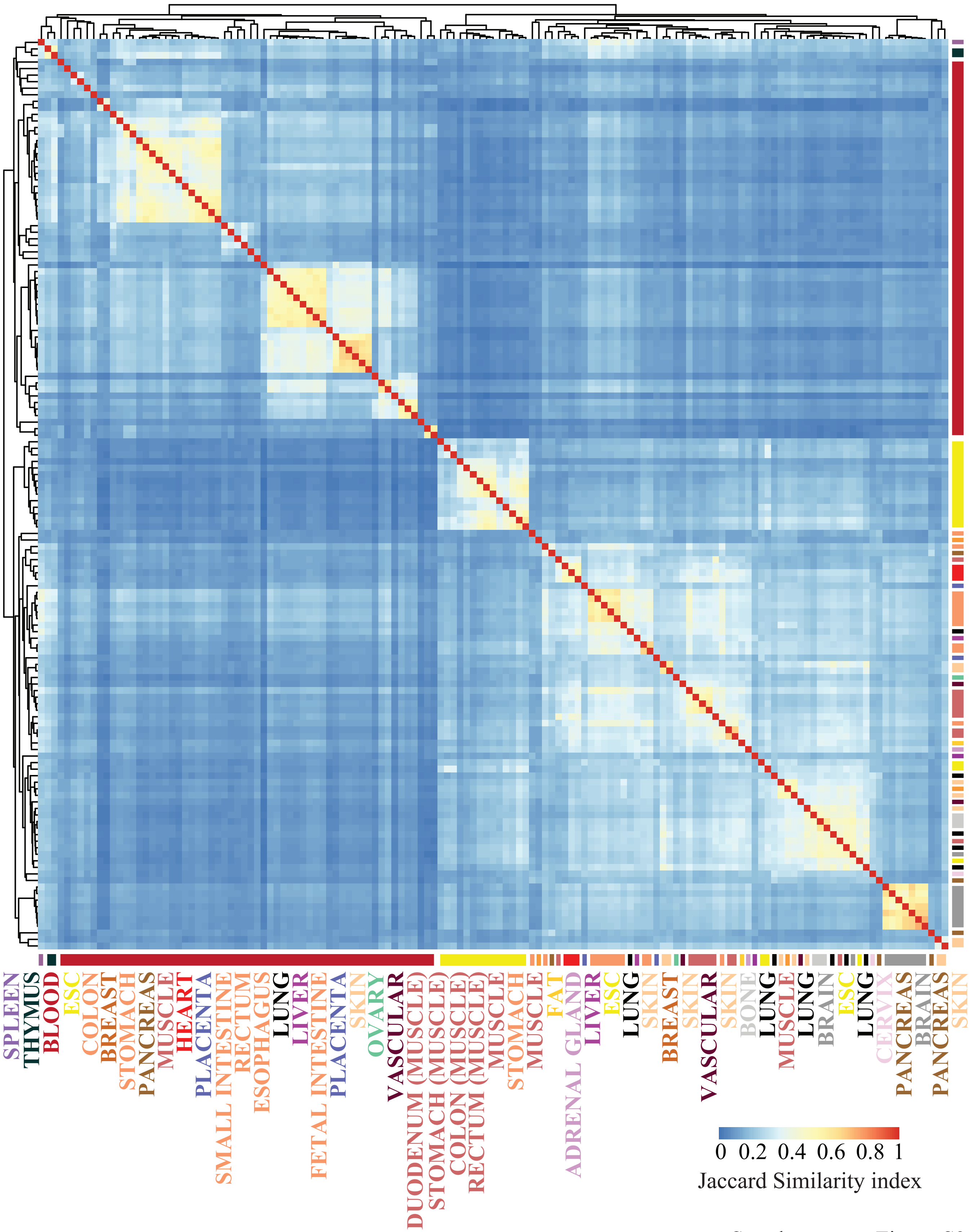

Supplementary Figure S3

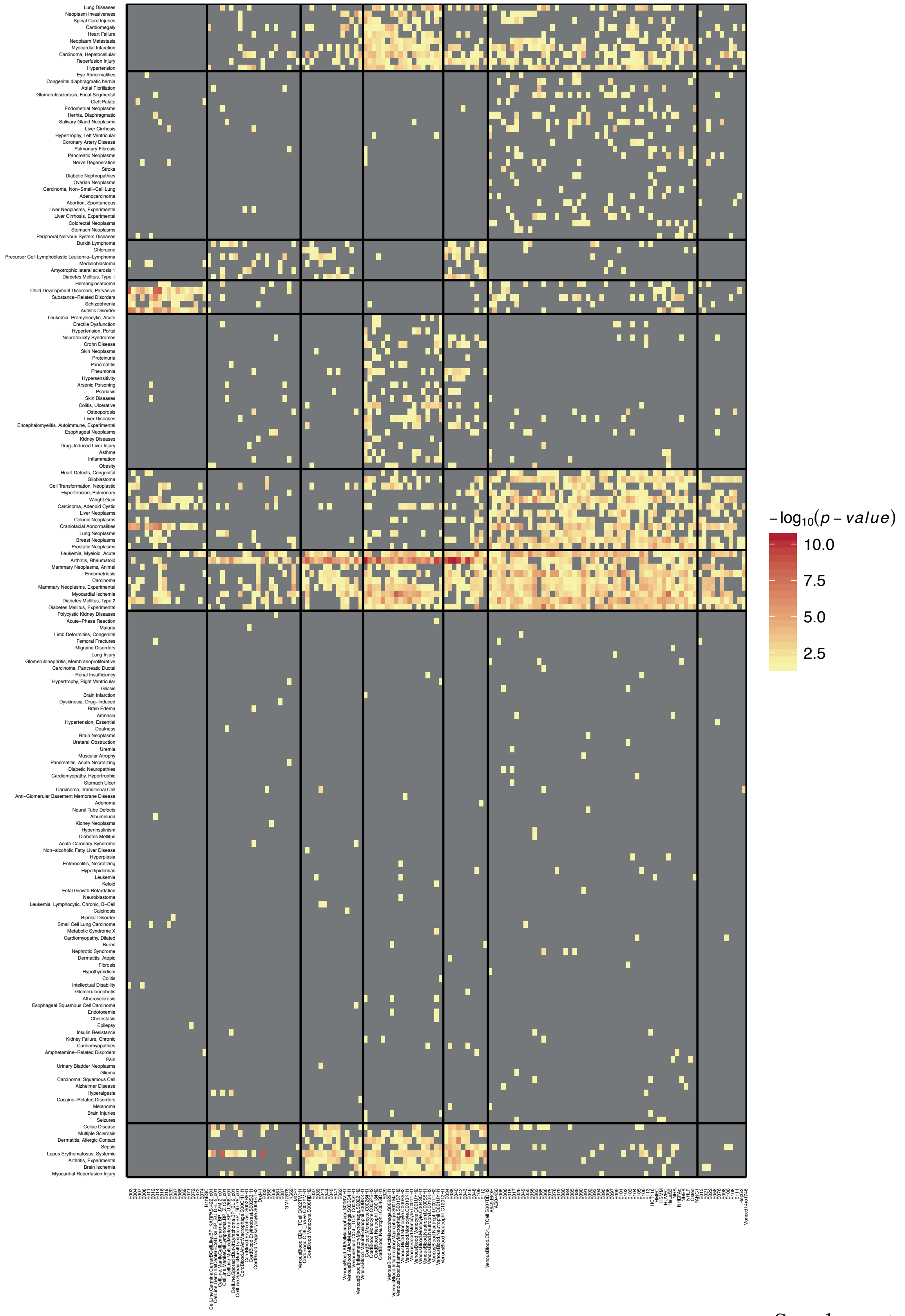

Supplementary Figure S4

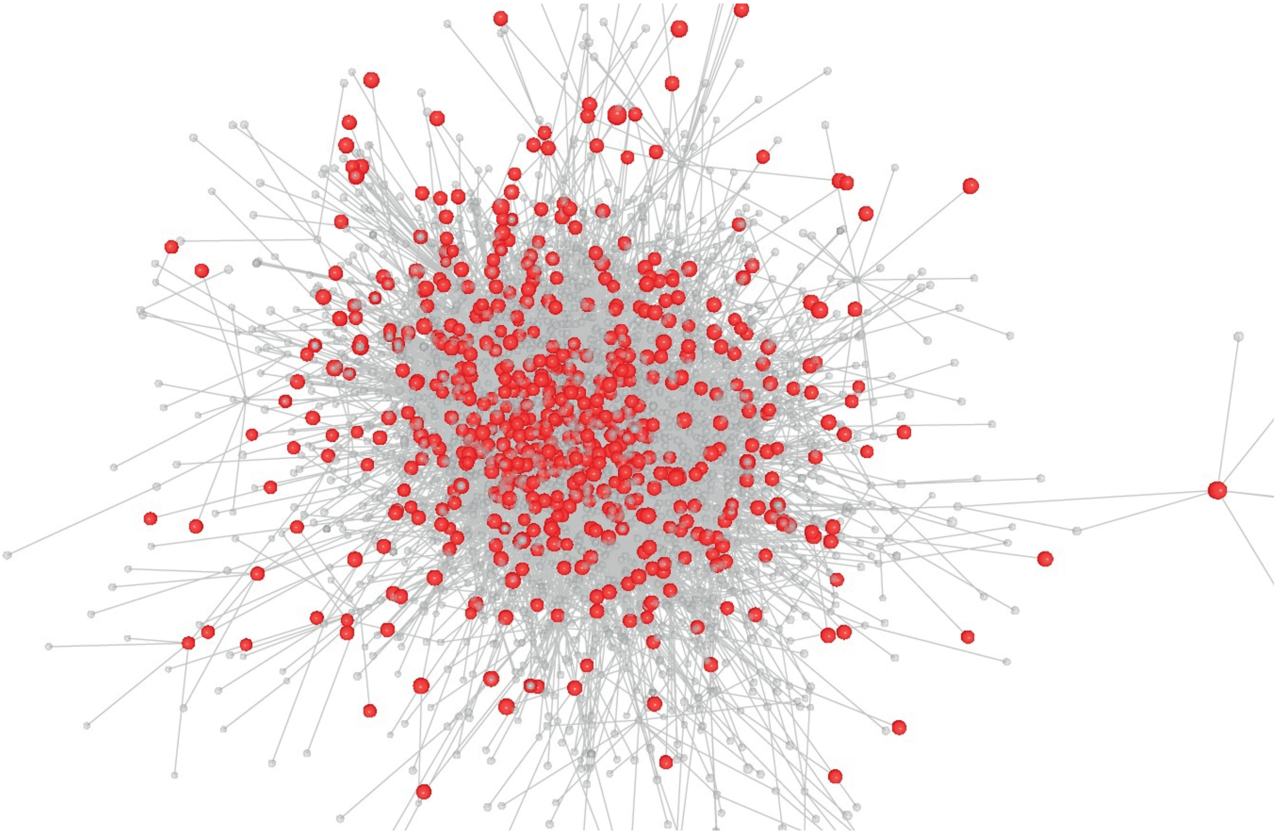

Supplementary Figure S5

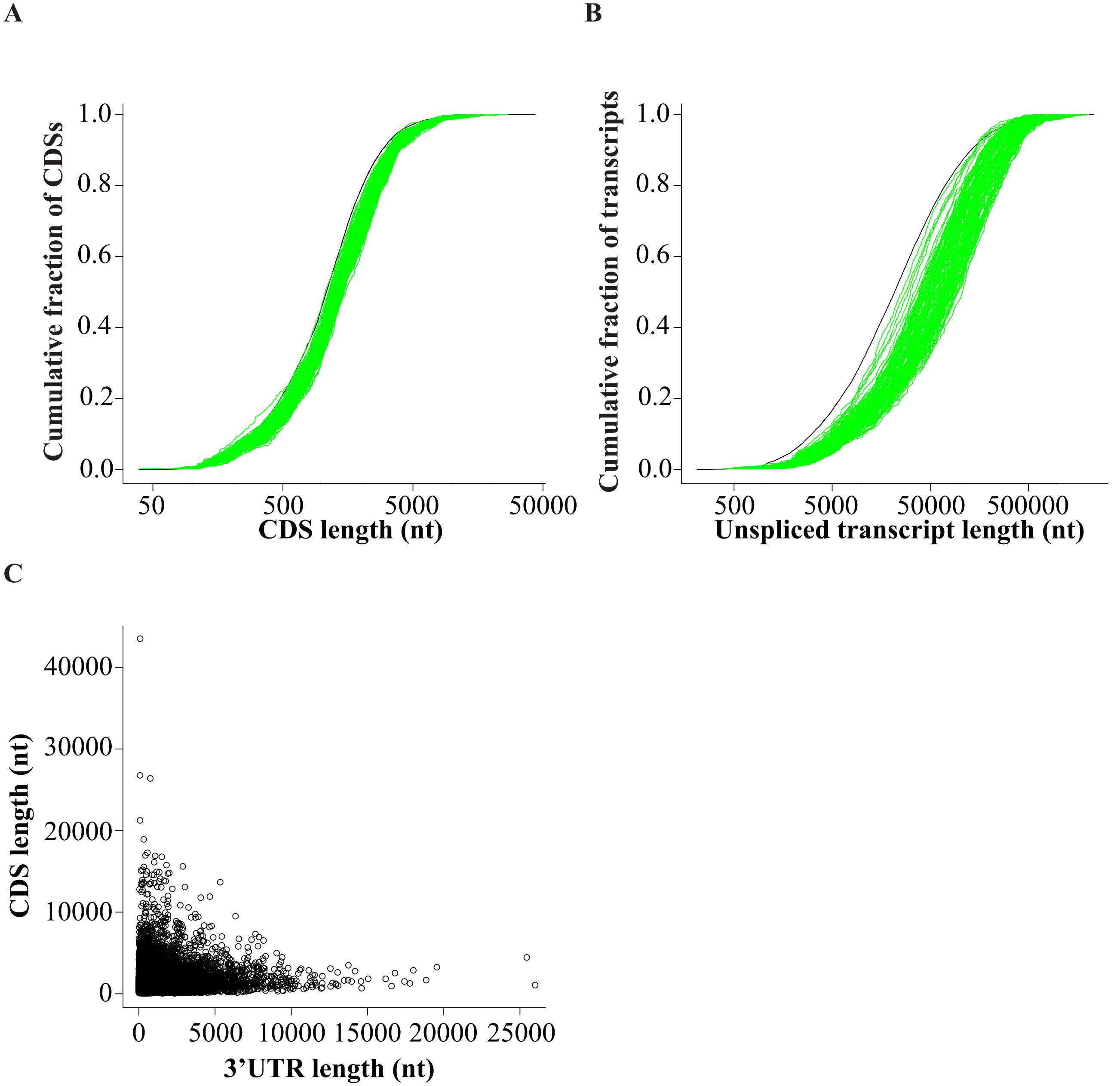

Supplementary Figure S6
