# Supplementary figures and images for: Cell type-selective disease-association of genes under high regulatory load
Source: Nucleic Acids Res. 2015 Oct 10;43(18):8839–55. doi: 10.1093/nar/gkv863 (PMC4605313; doi:10.1093/nar/gkv863)

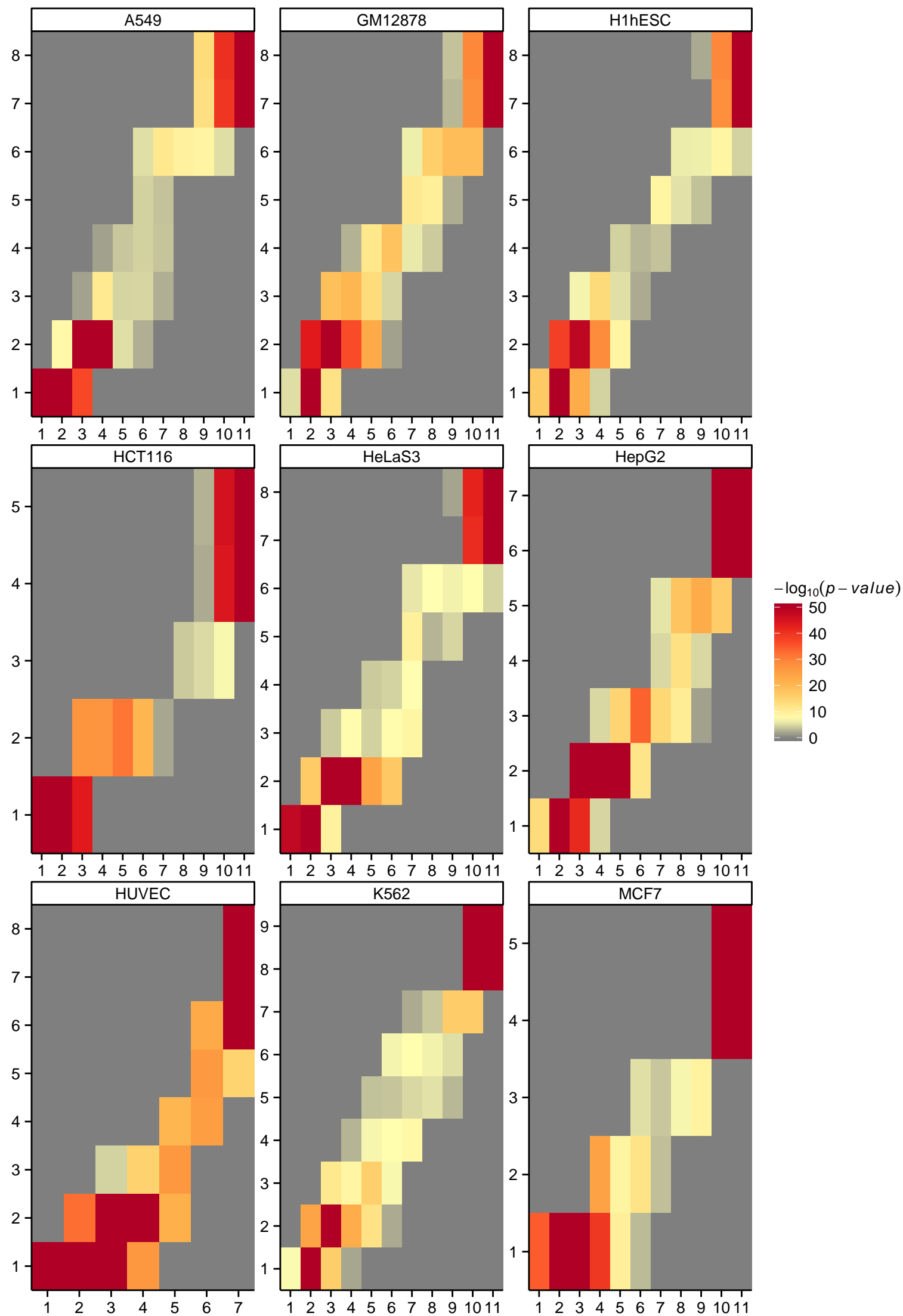

Supplement: SUPPLEMENTARY DATA [file supp_gkv863_nar-01077-z-2015-File013.zip › Galhardo_et_al_SUPPLEMENTARY_FILE_5/HeatmapTFs-vs-Enh_-log10pval.pdf]

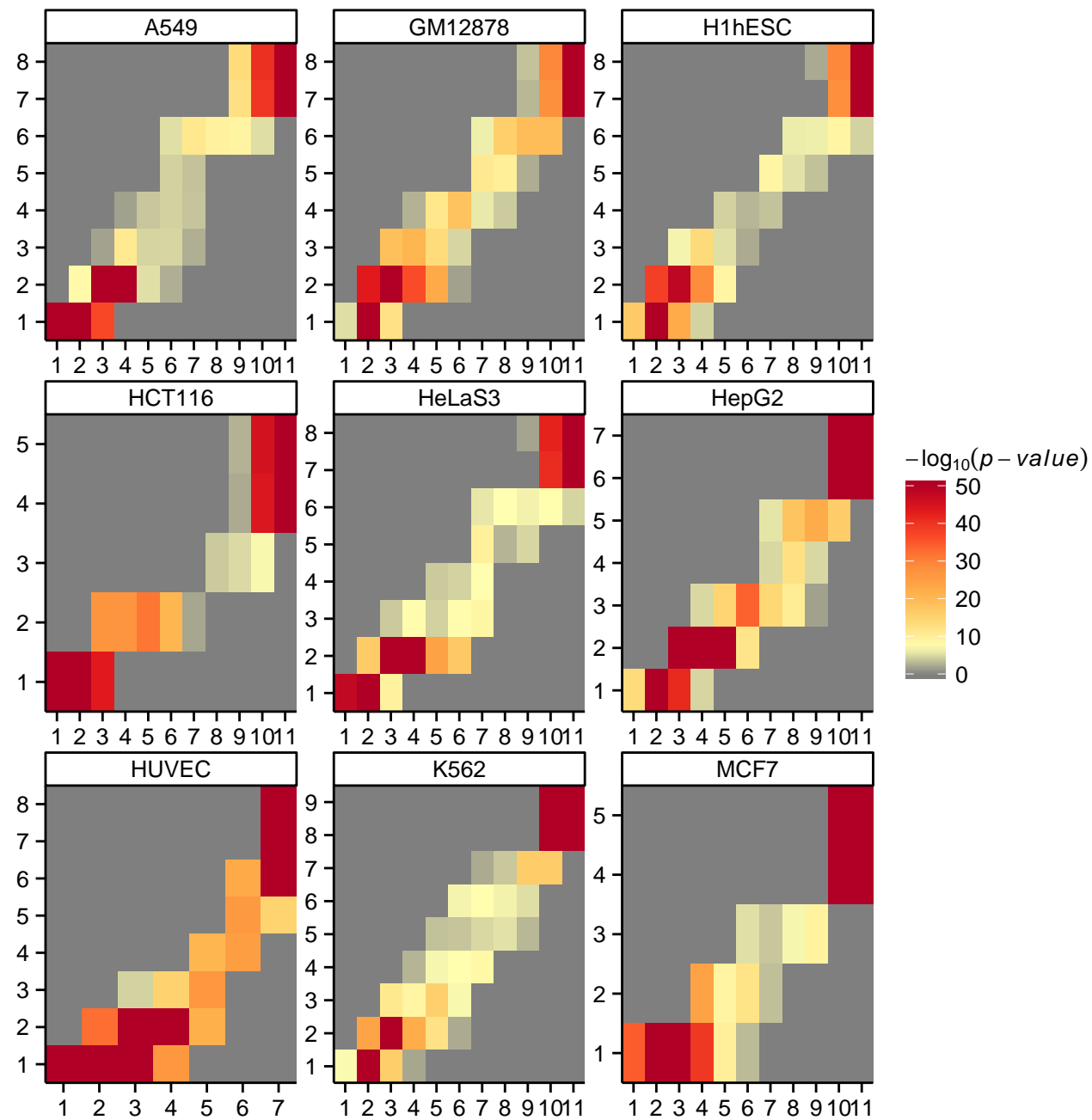

Supplement: SUPPLEMENTARY DATA [file supp_gkv863_nar-01077-z-2015-File013.zip › Galhardo_et_al_SUPPLEMENTARY_FILE_5/HeatmapTFs-vs-Enh_-log10pval2.pdf]
